# T Cell Exhaustion in Hepatocellular Carcinoma: A Substantial Barrier in Immunotherapy

**DOI:** 10.1111/jcmm.71044

**Published:** 2026-02-06

**Authors:** Kosar Nouri, Negar Asadollahei, Yasamin Haghir‐Sharif‐Zamini, Homeyra Seydi, Mahsa Salehi, Mehrnaz Mesdaghi, Mustapha Najimi, Massoud Vosough

**Affiliations:** ^1^ Department of Regenerative Medicine, Cell Science Research Center Royan Institute for Stem Cell Biology and Technology, ACECR Tehran Iran; ^2^ Biological Products and Blood Safety Research Center High Institute for Research and Education in Transfusion Medicine Tehran Iran; ^3^ Department of Allergy and Clinical Immunology Mofid Children's Hospital, Shahid Beheshti University of Medical Sciences Tehran Iran; ^4^ Department of Immunology, School of Medicine Shahid Beheshti University of Medical Sciences Tehran Iran; ^5^ Laboratory of Pediatric Hepatology and Cell Therapy Institute of Experimental and Clinical Research, UCLouvain Brussels Belgium; ^6^ Private University of Marrakesh Marrakesh Morocco; ^7^ Experimental Cancer Medicine, Institution for Laboratory Medicine Karolinska Institute Stockholm Sweden; ^8^ Department of Medical Biotechnology, School of Advanced Technologies in Medicine Tehran University of Medical Sciences Tehran Iran

**Keywords:** epigenetic modifications, hepatocellular carcinoma, signalling pathways, solid tumour immunotherapy, T cell exhaustion, tumour metabolomics

## Abstract

Hepatocellular carcinoma (HCC), accounting for over 90% of primary liver cancers, remains a major global challenge for healthcare professionals. While immunotherapy has transformed the landscape of cancer treatment, its success is often limited by immune resistance, particularly through T cell exhaustion which remains a major barrier to effective immune responses in solid tumours, including HCC. As tumours progress, T cells undergo a gradual loss of functionality due to continuous antigen exposure and fail to exert effective anti‐tumour responses. During this process, alterations in the epigenome, transcriptome, signalling pathways, and tumour metabolome, in addition to interactions with other cells in the tumour microenvironment, efficiently contribute to T cell exhaustion. Restoring T cell function brings hope for improving therapy outcomes and providing new treatment modalities for HCC patients. In this review, we explore the key cellular and molecular mechanisms driving T cell exhaustion, including the roles of immunosuppressive cells, metabolic stress, and epigenetic alterations focusing on HCC. We also discuss current and emerging strategies aimed at preventing or reversing T cell exhaustion, such as epigenetic modulation, immune checkpoint blockade, metabolic reprogramming, and combination therapies. Understanding these interconnected pathways is critical for designing more effective immunotherapy‐based approaches for liver cancer.

## 1 Introduction

Hepatocellular carcinoma (HCC) is the most common type of primary liver cancer, and the third most fatal cancer, accounting for 757,948 deaths in 2022 [1]. While our understanding of cancer aetiology, including genomic and microenvironment factors, is improving, the majority of patients are still diagnosed at an advanced stage, with less than 30% eligible for curative treatment options [2]. This highlights the urgent need for effective systemic therapies for the majority of HCC patients with advanced diseases. Immunotherapy provides an effective and safe approach for treating solid tumours, leading to prolonged survival and manageable side effects [3].

Immune checkpoint blockade and chimeric antigen receptor (CAR)‐T cell therapy are two strategies that have revolutionised cancer treatment [4]. Immunotherapy has been effective in more than 60% of haematological malignancies, such as leukaemia and lymphomas [5]; however, it shows limited efficacy in solid tumours [4, 6]. Moreover, despite success in relapsed and refractory B‐cell malignancies, CAR‐T cell therapies have not produced durable responses in patients with solid tumours, underscoring the need to enhance immunotherapy efficacy [7, 8, 9, 10]. Tumour‐infiltrating lymphocytes (TILs), among them CD8^+^ T cells, are the primary defenders against tumour cells [11]. Thus, the higher levels of TILs correlate with longer overall survival [12]. Although TILs are abundant in HCC tumour samples, their effectiveness in antitumor activity is surprisingly limited [13, 14]. The efficacy of CD8^+^ T cells in tumour elimination is significantly determined by their proliferation and functional performance [12]. Indeed, ongoing T‐cell stimulation by persistent tumour antigens and/or immunosuppressive tumour microenvironment (TME) leads to a dysfunctional state known as T‐cell exhaustion [15, 16]. Naïve T cells, upon encountering an antigen, differentiate into effector T (T_EFF_) cells. However, the proliferation and activity of some of these effector cells may diminish. This reduced responsiveness is not necessarily a failure of the immune system; it may play a protective role by preventing excessive immune reactions. The unresponsive T cells can be categorised into three main states: tolerance, anergy, and exhaustion [17]. Exhausted T (T_EX_) cells exhibit reduced proliferative capacity, diminished effector functions, impaired differentiation into memory T (T_MEM_) cells, and deficient metabolic activity, coupled with an increase in immune inhibitory receptors [18, 19]. The interaction between the inhibitory receptors on T cells and their ligands on tumour cells transmits a potent ‘do‐not‐eat‐me’ signal, hindering the clearance of tumour cells. This mechanism represents a crucial strategy employed by tumour cells to evade immune detection and destruction [12, 20]. General inhibitory receptors in HCC include programmed cell death protein 1 (PD‐1), T cell immunoglobulin and mucin‐domain containing‐3 (TIM‐3), lymphocyte activation gene 3 (LAG‐3), and cytotoxic T‐lymphocyte‐associated protein 4 (CTLA‐4) [21].

Hence, investigating the mechanisms and signalling pathways that drive T‐cell exhaustion could significantly aid in the design of treatment approaches to improve the immune response in the TME [13]. Progenitor exhausted T (T_PEX_) cells have the potential to differentiate into fully functional effector‐exhausted CD8^+^ T cells [22], and display memory‐like features, thereby providing a long‐term and superior response against tumour cells [23]. The aim of immunotherapies in this context is to promote exhausted T cells toward T_PEX_ cells with effector potential [22] and a memory‐like phenotype with superior proliferative capacity to achieve a durable response [24]. This review explores different mechanisms contributing to T cell exhaustion including epigenetic alterations, diverse signalling pathways, metabolic reprogramming, and the roles of different cells within the TME. It also discusses the therapeutic strategies to mitigate T‐cell exhaustion and enhance the anti‐tumour immunity in solid tumours, with a particular focus on HCC.

## 2 Epigenetic Reprogramming and Chromatin Remodelling in Exhausted CD8 ^+^ T Cells: Implications for HCC Immunotherapy

Studies have shown that the epigenetic profile of exhausted CD8^+^ T cells is distinct from that of effector or memory cells [25, 26]. Epigenome regulation is governed by the interplay between two key components: cis‐acting DNA elements, such as enhancers, and trans‐acting transcription factors (TFs) [27]. Substantial epigenetic reprogramming in CD8^+^ T cells, including DNA methylation, histone modifications, and alterations in chromatin architecture, contributes to the development of exhaustion [27, 28].

### 2.1 Targeting Epigenetic Regulators to Mitigate T Cell Exhaustion in HCC

**FIGURE 1:**
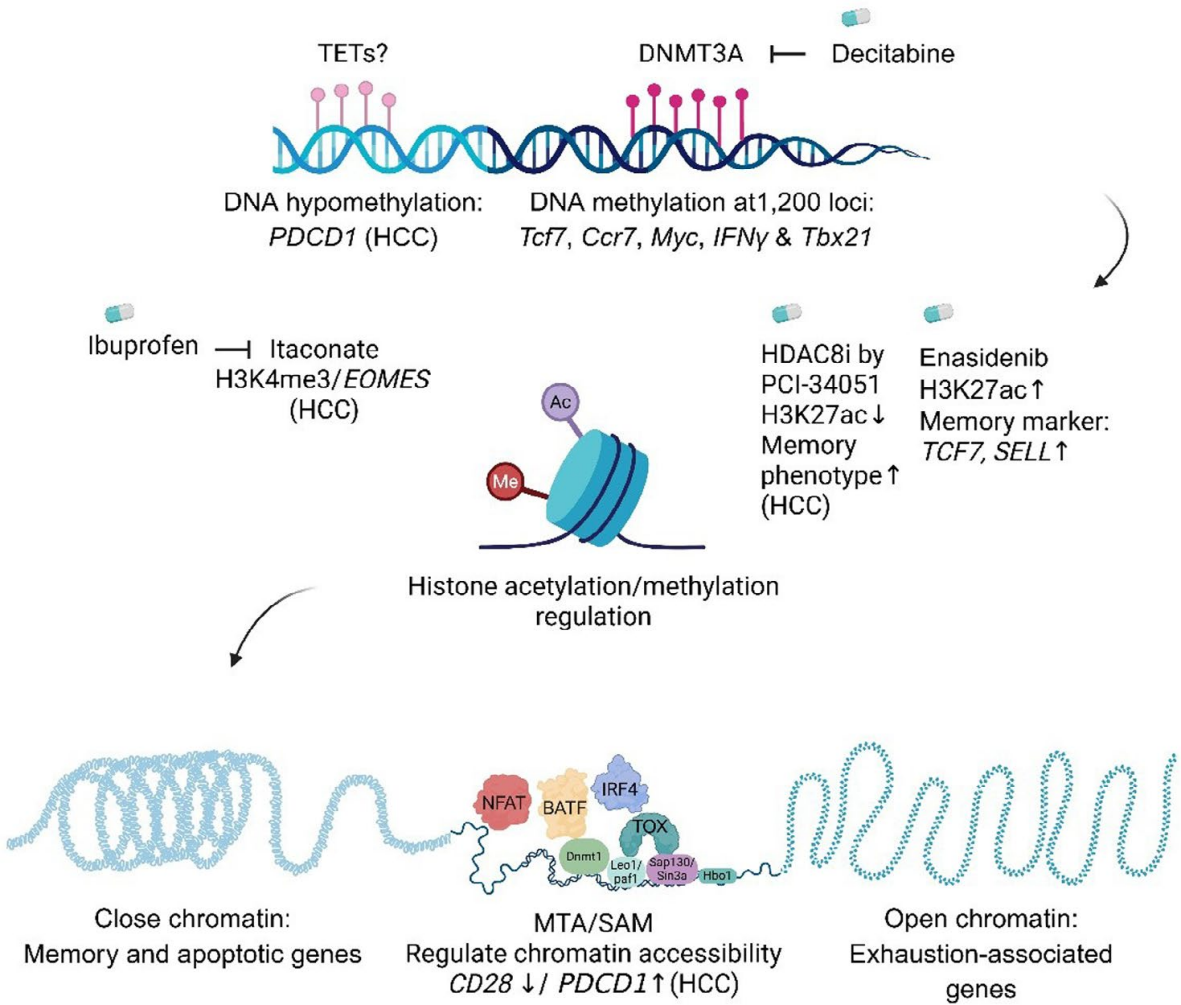
Epigenetic regulation of T cell exhaustion and therapeutic possibilities. DNA methylation profiling of peripheral blood mononuclear cells (PBMCs) from hepatocellular carcinoma (HCC) patients reveals that loci such as programmed cell death 1 (*PDCD1*) become hypomethylated with disease progression. DNA methyltransferase 3A (DNMT3A) mediates approximately 1200 de novo DNA methylation events at specific loci repressed during T cell exhaustion, including T‐cell factor 7 (*Tcf7*), C‐C motif chemokine receptor 7 (*Ccr7*), Myc proto‐oncogene (*Myc*), interferon gamma (*IFN‐γ*), and T‐box transcription factor 21 (*Tbx21*) in PD‐1^hi^ CD8^+^ T cells from a murine prostate tumour model. Decitabine (5‐aza‐2′‐deoxycytidine) enhances tumour‐infiltrating lymphocyte (TIL) proliferation and reduces tumour growth in vivo. Active and passive chromatin marks generate open or closed conformations that modulate gene expression to promote T cell exhaustion. Itaconate, a tricarboxylic acid (TCA) cycle metabolite, promotes exhaustion via histone H3 lysine 4 trimethylation (H3K4me3) at the eomesodermin (*EOMES*) locus, whereas ibuprofen reduces itaconate production and mitigates exhaustion. Inhibition of histone deacetylase 8 (HDAC8) increases histone H3 lysine 27 acetylation (H3K27ac), thereby enhancing the memory phenotype in an HCC model. Enasidenib, an isocitrate dehydrogenase 2 (IDH2) inhibitor, increases H3K27ac and transcription of memory‐associated genes *Tcf7* and L‐selectin (*SELL*). Various transcription factors cooperate with chromatin remodelers to promote the expression of exhaustion‐related genes while repressing memory and effector programs. Thymocyte selection‐associated high mobility group box (TOX) exerts regulatory functions, in part by interacting with histone acetyl transferase binding to origin recognition complex subunit 1 (HBO1), thereby promoting histone H4 and H3 acetylation. Additionally, TOX associates with proteins that mediate closed or open chromatin states, including DNA methyltransferase 1 (DNMT1), LEO1, a subunit of the RNA polymerase II–associated factor 1 (PAF1) complex, and Sin3A‐associated protein 130/Switch‐independent 3A transcription regulator (SAP130/SIN3A). In T_EX_ cells, probably together with the interferon regulatory factor 4–basic leucine zipper ATF‐like transcription factor (IRF4: BATF) complex, TOX regulates over 300 genes, leading to upregulation of genes such as T cell immunoglobulin and mucin‐domain containing‐3 (*Havcr2*, encoding Tim‐3), T‐box transcription factor 21 (*Tbx21*), interferon gamma (*Ifn‐γ*), cytotoxic T‐lymphocyte associated protein 4 (*Ctla4*), *Pdcd1*, T cell immunoreceptor with Ig and ITIM domains (*Tigit*), and *Tox* itself, while downregulating genes like interferon regulatory factor 8 (*Irf8*), B‐cell lymphoma/leukaemia 11B (*Bcl11b*), and interleukin‐10 (*Il10*). Furthermore, TOX suppresses apoptotic genes, including Fas cell surface death receptor (*Fas*), tumour necrosis factor (*Tnf*), growth arrest‐specific 2 (*Gas2*), and TNF receptor superfamily member 25 (*Tnfrsf25*) during continuous antigen exposure. Assay for transposase‐accessible chromatin using sequencing (ATAC‐seq) analysis shows that 5‐methylthioadenosine (MTA) and S‐adenosylmethionine (SAM) reduce chromatin accessibility at the CD28 locus while increasing accessibility near PDCD1 in HCC. Created in https://BioRender.com.

Peripheral immune cells in HCC patients exhibit distinct DNA methylation alterations compared with those in healthy individuals, reflecting tumour‐associated immune remodelling. Notably, DNA methylation profiling of peripheral blood mononuclear cells (PBMCs) from HCC patients reveals that some loci, such as programmed cell death 1 (*PDCD1*), become hypomethylated with HCC progression [29]. In contrast, during exhausted T‐cell differentiation, DNA methyltransferase 3A (DNMT3A) mediated approximately 1200 *de novo* DNA methylation events at loci such as T cell factor 7 (TCF7), C‐C chemokine receptor 7 (CCR7), MYC proto‐oncogene, bHLH transcription factor (MYC), interferon gamma (*IFN‐γ*), and T‐box transcription factor 21 (TBX21) in PD‐1^hi^ CD8^+^ T cells from a murine prostate tumour model. This methylation, which resists PD‐1 blockade, could be modulated by pretreatment with decitabine (5‐aza‐2′‐deoxycytidine), leading to enhanced TILs' proliferation and reduced tumour growth in vivo [30]. DNA methylation often reduces gene expression by directly blocking the binding of specific TFs. Additionally, it indirectly facilitates the recruitment of proteins that remodel chromatin into a repressed conformation [31]. Both activating and repressive histone modifications recruit chromatin remodelers that regulate transcription by modulating chromatin structure [32, 33]. Active chromatin marks, such as histone H3 trimethylated at lysine 4 (H3K4me3), histone H3 acetylated at lysine 27 (H3K27ac), and H3K9ac, and passive marks, such as H3K27me3, regulate gene expression during the differentiation from progenitor to terminally exhausted state. For instance, macrophage‐derived itaconate, a tricarboxylic acid (TCA) cycle metabolite, inhibits succinate dehydrogenase (SDH) in CD8^+^ T cells, driving exhaustion via the H3K4me3/eomesodermin (*EOMES*)/*PDCD1*/hepatitis A virus cellular receptor 2 (*HAVCR2*) axis. Ibuprofen reduces itaconate production by inhibiting nuclear factor kappa‐light‐chain‐enhancer of activated B cells (NF‐κB), thereby mitigating T cell exhaustion [11]. In contrast, the addition of repressive H3K27me3 marks leads to the downregulation of progenitor‐specific genes such as *TCF7*. Conversely, removal of repressive marks is required to upregulate genes associated with terminal exhaustion, including thymocyte selection‐associated high mobility group box (TOX) [34]. This highlights the potential for epigenetic drugs (epi‐drugs) to modify these epigenetic alterations reversibly. Treatment with a histone deacetylase 8 (HDAC8) inhibitor increases H3K27 acetylation, enhancing the number of CD8^+^ T cells within the TME. In addition, it promotes a memory phenotype and improves tumour control in an HCC model when used with anti‐programmed cell death ligand‐1 (PDL‐1) [35]. Enasidenib, an inhibitor of isocitrate dehydrogenase 2 (IDH2), enhances H3K27ac and transcription of memory marker genes, *TCF7* and L‐selectin (*SELL*) [36]. Conversely, enhancer of zeste homologue 2 (EZH2) preferentially methylates H3K27 at these regions [37]. Tazemetostat (Taz) is an S‐adenosylmethionine (SAM) competitive inhibitor of EZH2. Temporary inhibition of EZH2 using Taz maintains stemness and polyfunctionality in T cells while decreasing the level of inhibitory receptors such as PD‐1, LAG‐3, and TIM‐3. Notably, permanent EZH2 deletion results in limited tumour control in murine melanoma and human leukaemia [37]. Under hypoxic conditions within the TME, *EZH2* expression remains sustained, while the expression of *KDM6B*, histone H3 lysine 27 demethylase, decreases, indicating an increase in chromosome bivalency. Consequently, the overexpression of *KDM6B*, which is less sensitive to oxygen levels than *KDM6A*, effectively restores the expression of genes associated with T cell functionality (interleukin 2, *IL‐2*), stemness (B‐cell lymphoma 6 protein, *BCL6*), and the memory phenotype (SLAMF6) [34]. Tumour‐derived metabolites could also influence chromatin accessibility. An enhanced methionine salvage pathway ratio in HCC correlates with increased expression of exhaustion markers (e.g., *PDCD1*, *HAVCR2*) and reduced expression of T cell activation genes (e.g., C‐X‐C motif chemokine receptor 3/6, *CXCR3/6*, *CCR7*). Metabolites like 5‐methylthioadenosine (MTA) and SAM decrease IFN‐γ secretion while elevating TOX, PD‐1, and TIM‐3 [38]. SAM contributes to epigenetic modifications by acting as a methyl donor for DNA and protein methyltransferases [39]. Transposase‐accessible chromatin with high‐throughput sequencing (ATAC‐seq) analysis shows that MTA and SAM reduce *CD28* chromatin accessibility while increasing it near the *PDCD1* locus [38] (Figure 1). These findings highlight the potential of targeting epigenetic regulators to reverse T cell exhaustion and enhance immune responses in HCC.

### 2.2 Targeting T_PEX_ to Overcome Epigenetic Barriers in Checkpoint Blockade Immunotherapy

Chronic antigen stimulation causes stable epigenetic scars that limit T cell reinvigoration, which persists even after PD‐1 blockade. Among 6000 unique open chromatin regions (OCRs) distinguishing T_EX_ from T_EFF_ or T_MEM_ state, only 650 OCRs were altered by PD‐1 blockade, reflecting the stability of exhaustion‐associated epigenetic programs [27, 40, 41]. In an HCC model, dysfunctional T cells display heterogeneous reinvigoration capacity. Two PD‐1^hi^ subsets were identified: early‐stage T_EX_ cells (day 5, CD38^lo^/CD101^lo^), which respond to IL‐15, and late‐stage T_EX_ cells (post‐day 12, CD38^hi^/CD101^hi^), which fail to recover due to fixed chromatin landscapes, as shown by ATAC‐seq [42].

Not all exhausted T cells are equally affected by PD‐1 blockade. T_PEX_ cells show epigenetic plasticity, with accessible chromatin related to stemness and memory (*Tcf7*, *Bcl6*, inhibitor of DNA binding 3, *Id3*), suggesting that exhaustion can be reinvigorated in this subset upon PD‐1 blockade [8, 21, 25, 43, 44, 45, 46]. In contrast, in the T_EX_ state, the chromatin accessibility of stem and effector genes was diminished, while exhaustion‐related genes (*TOX*, *PDCD1*, *HAVCR2*) gain accessibility [25]. With ~80% ineffective rate of checkpoint inhibitors in HCC patients [21, 47], expanding or preserving T_PEX_ cells offers a promising strategy to restore antitumor immunity and improve immunotherapy outcomes [48].

### 2.3 Targeting TOX to Modulate T Cell Exhaustion: A Chromatin‐Based Approach to Enhance Antitumor Immunity in HCC

TOX contributes to chromatin accessibility of approximately 4000 regions in the genome [49]. TOX is a high‐mobility group box protein that functions by binding to DNA based on its structure, rather than a specific motif sequence [50]. It exerts its regulatory effects partly by interacting with the histone acetyl transferase binding to origin recognition complex subunit 1 (HBO1), thereby promoting histones H4 and H3 acetylation. Additionally, TOX associates with proteins that mediate closed/open epigenetic alterations, such as DNMT1, a subcomplex of the RNA polymerase II‐associated factor 1 complex (Paf1C) (Leo1)/polymerase‐associated factor 1 (Paf1), Sap130/Sin3A: Sin3A‐associated protein 130/Switch‐independent 3A transcription regulator (Sap130/Sin3a) (Figure 1) [49].

In HCC, TOX expression is notably upregulated in a subset of exhausted T cells within the *Layilin* (*Layn*) cluster [49]. Studies using an autochthonous liver cancer model demonstrate that TOX expression increases and remains elevated during tumour progression and becomes restricted to T_EX_ cells in HCC patients [49, 50]. Consistent with ATAC‐seq analysis, *TOX*‐knockout prevents the upregulation of inhibitory receptors in liver cancer, although effector function declines similarly to that of wild‐type T cells. This indicates that regulation of inhibitory receptors may occur independently of impairment of effector function [50]. Further chromatin accessibility analysis reveals that *TOX* deletion reduces accessibility at loci such as *Tox*, *Pdcd1*, *Cd38*, and ectonucleoside triphosphate diphosphohydrolase 1 (*Entpd1*), while increasing accessibility at *Tcf7*, *Cd28*, fyn proto‐oncogene (*Fyn*), and interleukin 7 receptor (*Il7r*) [50]. By contrast, results show that *TOX* ^*−/−*^ leads to a closed chromatin conformation in T_MEM_ and T_PEX_ progenitors, affecting genes like *Tcf7*, *Ccr7*, *Slamf6*, BTB domain, and CNC homology 2 (*Bach2*), and IKAROS family zinc finger 2 (*Ikzf2*) in chronic infection [49].

T cell exhaustion appears to serve as a mechanism to prevent apoptosis and terminal differentiation under conditions of persistent stimulation [51]. Notably, complete deletion of genes such as *PD‐1*, *TOX*, and interferon regulatory factor 4 (*IRF4*) significantly reduces the antigen‐specific T cell populations [49, 50, 52, 53]. However, partial reduction of *TOX* by heterozygous deletion enhances T cell proliferative capacity and improves tumour control, supporting the notion that controlled reduction of *TOX* expression could represent a promising therapeutic approach to reinvigorate T cell responses and enhance the efficacy of cancer immunotherapy [49].

## 3 Genetic and Molecular Profiles of Exhausted T Cells in HCC

As discussed earlier, inhibitory receptors (as markers of exhausted T cells) are highly expressed in both preclinical and clinical HCC tumours [11, 21, 54]. Furthermore, the recognised exhaustion markers *ENTPD1*, T cell immunoreceptor with Ig and ITIM domains (*TIGIT*), tumour necrosis factor receptor superfamily member 9 (*TNFRSF9*), and *CD27* (encodes TNFRSF7) have been demonstrated in TILs from HCC samples that show higher levels as the disease progresses to later stages [11]. Zheng and his colleagues also introduced less‐discussed exhaustion‐associated genes, such as myosin VIIA (*MYO7A*), tryptophanyl‐tRNA synthetase (*WARS*), and C‐X‐C motif chemokine ligand 13 (*CXCL13*), as well as novel candidates such as *LAYN*, *PHLDA1*, and synaptosomal‐associated protein 47 kDa (*SNAP47*) [13]. A complex transcriptional network underlies the differentiation of T cells toward an exhausted phenotype. Alterations in the expression of key TFs such as Tox, Tbx21, EOMES, basic leucine zipper activating transcription factor‐like transcription factor (*BATF*), *IRF4*, nuclear factor of activated T cells 1 (*NFATC1*), *c‐MAF*, von hippel–lindau tumour suppressor (Vhl), and forkhead box O1 (Foxo1) have been reported in T_EX_ cells derived from HCC samples [11]. The following sections discuss the roles of these TFs in T cell exhaustion, specifically within the context of HCC and chronic antigen exposure.

### 3.1 TOX

TOX, a prominent TF involved in the differentiation process toward the exhaustion state, exhibits distinct target preferences across different cellular contexts [34]. Its expression increases in chronic viral infections and is observed in exhausted TILs isolated from various tumour samples, including HCC [13, 51, 55]. TOX enhances the PD‐1 stability by promoting its endocytic recycling. Downregulation of TOX alleviates T cell exhaustion and increases CD8^+^ TILs, thereby improving responsiveness to anti‐PD‐1 therapy in preclinical models of HCC [16]. In T_PEX_ cells, TOX binds to several genes, including *Tcf7*, lymphoid enhancer‐binding factor 1 (*Lef1*), *Bcl6*, *Bach2*, *Foxo1*, and *Id3*, leading to their upregulation, while downregulating interleukin‐2 receptor alpha chain (*Il2ra*) and *Irf4*. In T_EX_ cells, probably together with the interferon regulatory factor IRF4: BATF, TOX regulates over 300 genes, such as *Havcr2* (encoding Tim‐3), *Tbx21*, *Ifn‐γ*, *Ctla4*, *Pdcd1*, *Tigit*, and even *Tox* itself, which are upregulated, along with downregulated targets like *Irf8*, *Bcl11b*, and *Il10* [34]. Furthermore, TOX was reported to prevent apoptotic gene expression, including *Fas*, *Tnf*, *Gas2*, and *Tnfrs25*, during continuous antigen exposure (Figure 1) [50]. This differential binding pattern underscores TOX's critical function in orchestrating the transcriptional landscape during T cell differentiation and exhaustion [34].

### 3.2 BATF

BATF, a member of the activator protein 1 (AP‐1)/activating transcription factor (ATF) superfamily, was identified as an exhaustion‐associated gene that efficiently predicts survival outcomes in patients with HCC. Recent qPCR analysis of 60 liver cancer samples indicates that *BATF* expression is markedly reduced in tumour tissues compared with normal tissues [12]. Furthermore, BATF negatively impacts the expression of exhaustion‐related TFs, such as *EOMES* [56]. BATF also increases the expression of genes related to the effector state, including *RUNX3*, killer cell lectin‐like receptor G1 (*KLRG1*), and *TBX21* by binding to their regulatory elements [57], thereby facilitating the differentiation of progenitor cells into effector CD8^+^ T cells in chronic viral infection [58]. In addition, BATF contributes to the closed chromatin remodelling of the *TOX* locus. In contrast, Erythroblast transformation‐specific (ETS), RUNT, bZIP, and IRF motifs become more accessible in BATF‐overexpressing CAR‐T cells [56]. BATF overexpression in CAR‐T cells promotes the effector state and induces a memory phenotype upon tumour re‐challenge [56, 59]. On the other hand, BATF knockout enhances central memory characteristics [57] and decreases the frequency of PD‐1^+^TIM3^+^/PD‐1^+^TOX^hi^ CAR‐T cells, maintaining them in a naïve‐like phenotype. These findings indicate that, unlike progenitor cells, BATF exhibits significantly higher DNA binding in terminally dysfunctional cells [34]. In fact, BATF is considered an “ambivalent” TF, capable of promoting exhaustion or effector function depending on the signalling pathways in the background [56].

### 3.3 T‐bet and EOMES

EOMES and its homologous T‐bet are both members of the T‐box TFs family [20, 60]. The T‐bet: EOMES ratio determines the fate of T cells. Effector cells with the expression pattern of T‐bet^++^EOMES^+^ differentiate into terminal effector cells, while T‐bet^+^EOMES^++^ effector cells differentiate into memory CD8^+^ T cells. T_EX_ cells are terminal effector cells that, under chronic antigen exposure, downregulate T‐bet and upregulate EOMES expression (T‐bet^+^EOMES^+++^) [17]. A population characterised by high EOMES and PD‐1 expression (EOMES^hi^PD‐1^hi^) shows a positive correlation with the severity of hepatitis C virus (HCV) infection [60]. Moreover, PD1^hi^ CD8^+^ TILs in HCC samples exhibit increased *EOMES* and decreased *T‐bet* expression [21]. Loss of both alleles of *EOMES* diminishes effector and exhaustion markers but increases stemness potential in CD8^+^ T cells. Nonetheless, reducing EOMES to the basal level by loss of one allele increases effector and stemness markers while decreasing exhaustion states [60]. In contrast, another study on HCC samples shows that EOMES levels in CD8^+^ T cells are significantly lower in tumour regions than in adjacent normal tissues. This finding was confirmed by RNA‐seq analysis across different stages of HCC in the cancer genome atlas (TCGA) cohort [20]. However, a TF is not determined solely by its expression level; its cellular location is also crucial for its proper function. Both T‐bet and EOMES repress *Pdcd1*, though with differing binding affinities. T‐bet exhibits a stronger binding affinity to its target site compared to EOMES. During chronic antigen exposure, T‐bet is prevented from entering the nucleus, allowing EOMES to exert weaker suppression of *Pdcd1*, thereby contributing to its upregulation. Upon PD‐1 blockade, T‐bet re‐enters the nucleus leading to a robust suppression of *Pdcd1* gene expression [61]. Notably, among T_EX_ populations, T‐bet^hi^PD1^mid^ respond more favourably to PD‐1 blockade than EOMES^hi^PD1^hi^ T cells [62]. Moreover, *T‐bet* upregulation increases the effector potential of CAR‐T cells against pancreatic tumour cells [63].

### 3.4 Other Transcription Factors

Nuclear factor of activated T cells (NFAT) plays a dual role in regulating T cell fate. When paired with AP‐1, it promotes effector gene expression, thereby enhancing T cell function. However, in the absence of AP‐1, NFAT homodimers drive exhaustion by activating inhibitory genes such as *PDCD1* (PD‐1), *HAVCR2* (TIM‐3), *LAG‐3*, and *TOX* [17, 50, 64]. Notably, other findings report that NFAT also interacts with IRF4 and BATF to form a complex that promotes exhaustion [52]. *NFATC1*, *IRF4*, and *BATF* are upregulated in PD‐1^hi^ CD8^+^ TILs from HCC tissues [21]. NFAT, with its partner IRF4, enhances the expression of PD‐1 and TOX [65]. At elevated levels, IRF4 interacts with other TFs in a triplicate complex (NFAT, IRF4, and BATF), increasing the expression of inhibitory receptors while suppressing *TCF‐1* and cytokine‐related genes [52]. During persistent infection, IRF4 promotes the expansion of exhausted T‐bet^−^EOMES^+^ T cells [61, 66]; however, modest IRF4 reduction enhances the population of TCF‐7^+^ T_MEM_ [66]. Altogether, the newly formed TF network that drives T cell exhaustion is activated by exhaustion‐associated signalling pathways under chronic antigen exposure, which will be explored in detail in the following section.

## 4 Signalling Pathways Involved in T Cell Exhaustion

**FIGURE 2:**
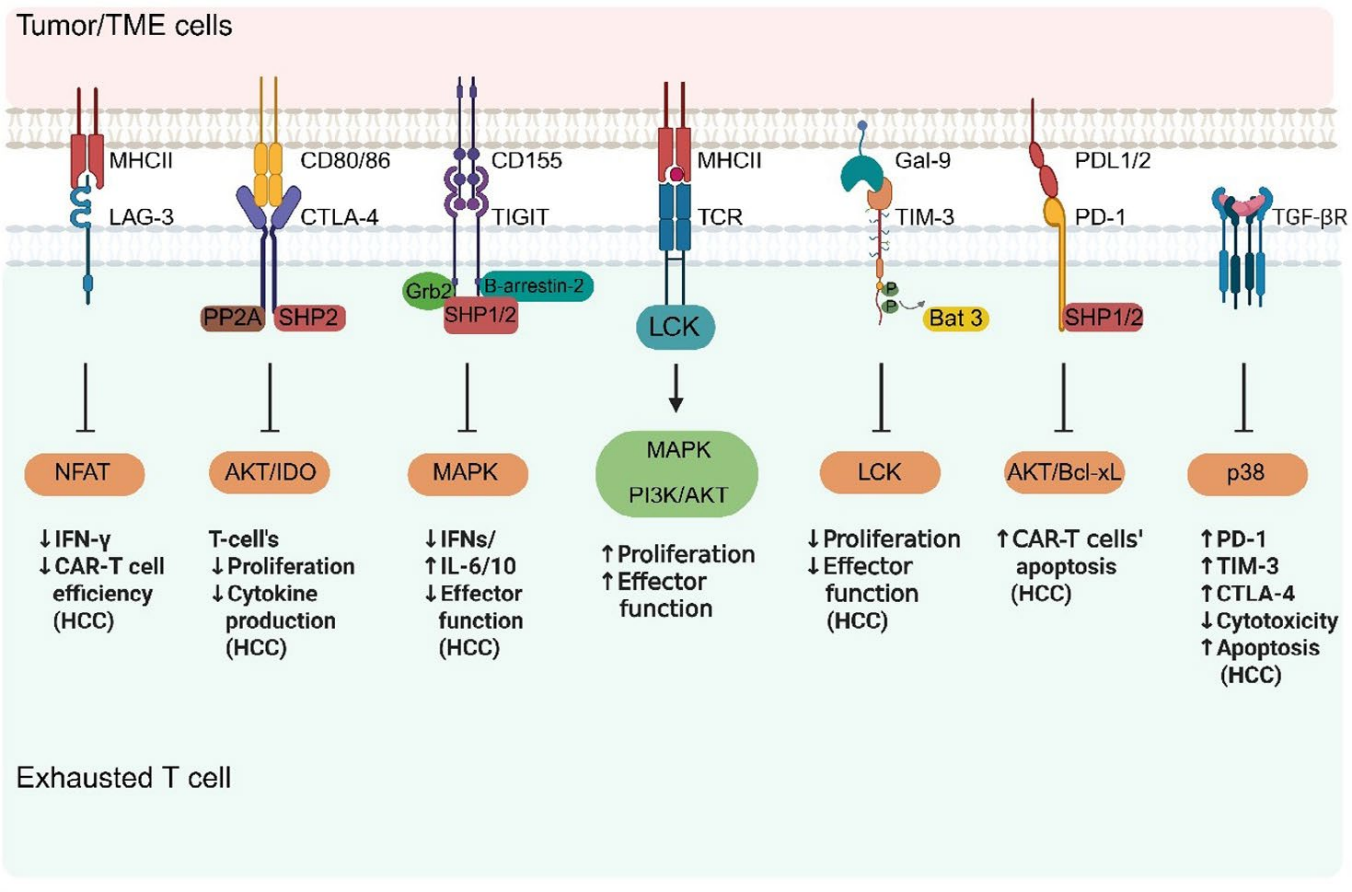
Inhibitory immune checkpoint signalling pathways lead to T‐cell exhaustion. Immune inhibitory receptors, including programmed cell death protein 1 (PD‐1), cytotoxic T‐lymphocyte‐associated protein 4 (CTLA‐4), T cell immunoreceptor with immunoglobulin and immunoreceptor tyrosine‐based inhibitory motifs domains (TIGIT), lymphocyte activation gene 3 (LAG‐3), and T cell immunoglobulin and mucin domain–containing protein 3 (TIM‐3), interact with their respective ligands on tumour and non‐tumour cells, resulting in T‐cell receptor (TCR)–mediated downstream signalling pathway suppression. TIGIT activation inhibits phosphoinositide 3‐kinase (PI3K)‐protein kinase B (PKB, also termed AKT) and mitogen‐activated protein kinase (MAPK) signalling pathways. CTLA‐4 inhibits PKB activity, which is essential for T‐cell proliferation, probably via its association with the serine/threonine phosphatase protein phosphatase 2A (PP2A). CTLA‐4 activates indoleamine 2,3‐dioxygenase (IDO), which degrades tryptophan and thereby mediates T‐cell proliferation. In the immunological synapse between an antigen‐presenting cell (APC) and a T cell, LAG‐3 binds to major histocompatibility complex class II (MHC class II, MHCII) and inhibits TCR signal transduction and interferon‐gamma (IFN‐γ) secretion in the activated peripheral blood mononuclear cells (PBMCs). PD‐1 activation leads to the phosphorylation of immunoreceptor tyrosine‐based inhibitory motif (ITIM) and immunoreceptor tyrosine‐based switch motif (ITSM) of the PD‐1 intracellular domain and recruitment of the tyrosine phosphatases Src homology phosphatase 1 and 2 (SHP‐1 and SHP‐2). This dephosphorylation inhibits downstream pathways, including phosphoinositide 3‐kinase (PI3K), protein kinase B (PKB)/Akt, mechanistic target of rapamycin (mTOR), and Ras/mitogen‐activated protein kinase/extracellular signal–regulated kinase (ERK). The PD‐1 pathway inhibits Akt and B‐cell lymphoma‐extra‐large (Bcl‐xL), allowing cells to evade apoptosis in hepatocellular carcinoma (HCC). Ligand binding to TIM‐3 leads to the release of HLA‐B–associated transcript 3 (BAT3) and facilitates tyrosine phosphorylation of the TIM‐3 tail, which then inhibits lymphocyte‐specific protein tyrosine kinase (LCK). In HCC, TIM‐3 reduces the proliferation capacity and effector function of T cells. Transforming growth factor‐beta 1 (TGF‐β1) increases the expression of PD‐1 and CTLA‐4, possibly through activation of the calcineurin–nuclear factor of activated T cells c1 (NFATc1) pathway, and TIM‐3 through the p38 mitogen‐activated protein kinase (p38 MAPK) signalling pathway in T cells in HCC. Created in https://BioRender.com.

In general, T cells are activated upon interaction between the T cell receptor (TCR) and peptide‐major histocompatibility complex (pMHC) ligands. The following signalling cascade includes the Src family kinase lymphocyte‐specific protein tyrosine kinase (LCK), which phosphorylates immunoreceptor tyrosine‐based activation motif (ITAM). The phosphorylated ITAM recruits and activates zeta‐chain‐associated protein kinase 70 (ZAP‐70), which then phosphorylates downstream adaptor proteins, such as linker for activation of T cells (LAT) and SH2 domain‐containing leukocyte protein of 76 kDa (SLP‐76). These adaptor proteins activate multiple downstream pathways, including phospholipase C gamma 1 (PLC‐γ1)‐dependent mobilisation of intracellular calcium, activation of protein kinase C (PKC), Ras‐MAPK, and phosphoinositide 3‐kinase (PI3K)‐protein kinase B (PKB, also termed AKT) pathways, ultimately promoting proliferation and cytokine production (Figure 2) [67, 68]. Although, during chronic antigen stimulation, T cells become exhausted, a state typically marked by increased expression of several inhibitory receptors, including PD‐1, CTLA‐4, B and T lymphocyte attenuator (BTLA), TIM‐3, LAG‐3, natural killer cell receptor 2B4 (2B4, CD244), and CD39, which are highly expressed in both preclinical and clinical HCC tumours and inhibit T cell activation [11, 21, 54]. Among the pathways contributing to this dysfunctional state, the PD‐1/PD‐L1 pathway stands out as the main one. It is closely linked to reduced T‐cell growth, cytokine release, and cytotoxicity. CTLA‐4 works alongside PD‐1 by blocking essential co‐stimulatory signals, such as CD28, while other checkpoints, including LAG‐3, TIM‐3, and TIGIT, add an extra layer of inhibition in the chronically antigenic TME. Beyond these surface checkpoints, broader immunosuppressive pathways, such as Wnt/β‐catenin and TGF‐β, also shape the TME by limiting T‐cell entry, increasing PD‐L1 levels, and dampening T‐cell activity, all of which help the tumour cells evade immune surveillance. These signalling pathways, together with other checkpoint inhibitors, cooperate, underscoring the promise of combination immunotherapies for HCC. In the following section, we discuss the contributions of these pathways to T‐cell exhaustion, focusing on HCC wherever possible (Figure 2).

### 4.1 PD‐1/PD‐L1 Pathway

PD‐1 is a primary immune checkpoint molecule expressed on both activated and exhausted T cells. During normal T cell activation, PD‐1 expression increases transiently. However, under chronic antigen exposure, it rises to high levels, leading to T‐cell exhaustion that inhibits proliferation and cytokine production. In addition, different TFs, including interferon regulatory factor 9 (IRF9), IFN‐α, FOXO1, and the AP‐1 subunit FBJ murine osteosarcoma viral oncogene homologue (c‐FOS), upregulate PD‐1 expression under chronic stimulation [69, 70]. Its ligand, PD‐L1, is expressed on various cell types, including tumour cells, immune cells, and endothelial cells [71], and is up‐regulated by INF‐γ secreted by TILs, leading to adaptive resistance to tumour‐specific antigen recognition [72]. PD‐L1 expression can also be enhanced by activating oncogenic signalling pathways such as Ras, mammalian target of rapamycin (mTOR), epidermal growth factor receptor (EGFR), mitogen‐activated protein kinase (MEK), extracellular signal‐regulated kinases (ERK), and MAPK [73]. PD‐1/PD‐L1 interaction triggers a signalling pathway in T cells, involving the phosphorylation of immunoreceptor tyrosine‐based inhibitory/switch motif (ITIM/ITSM) and the recruitment of tyrosine phosphatases the Src homology 2 domain‐containing phosphatases 1 and 2 (SHP‐1/2). This dephosphorylation inhibits downstream pathways, including PI3K, PKB/AKT, mTOR, and Ras/MAPK/ERK. This pathway contributes to transcriptional repression, T cell cycle arrest, reduced cytokine production, impaired proliferation and differentiation, functional exhaustion, and increased susceptibility to apoptosis [74, 75].

Clinical studies show that PD‐L1 is frequently overexpressed on HCC tumour cells and is linked to greater tumour aggressiveness, higher postoperative recurrence, and poorer overall survival [76, 77]. Therefore, PD‐1/PDL‐1 blockade therapy, which activates exhausted tumour‐infiltrating T cells, is a promising therapeutic option for HCC treatment [78]. Disruption of PD‐1 in the glypican‐3 (GPC3)‐targeted second‐generation CAR‐T cells increases AKT and Bcl‐xL levels, allowing cells to evade apoptosis in co‐culture with HCC cells (Figure 2) [79]. The main therapeutic approach to target the PD‐1/PD‐L1 axis is through different types of anti‐PD‐1 monoclonal antibodies. The first human immunoglobulin G 4 monoclonal antibody targeting PD‐1/PD‐L1 and PD‐L2 is nivolumab. In many clinical trials, it demonstrated good clinical efficacy and a safety profile, both as monotherapy and in combination with other therapeutic regimens, including other ICIs and/or TKIs [78]. Following the positive results of the phase III IMbrave150 trial, the United States Food and Drug Administration (FDA) approved atezolizumab (an anti–PD‐L1 monoclonal antibody) in combination with bevacizumab (an anti‐VEGF monoclonal antibody) as a first‐line treatment for patients with unresectable HCC [80]. As second‐line therapy, the FDA approved additional anti‐PD‐1 agents, nivolumab or pembrolizumab, for sorafenib‐pretreated patients with advanced HCC [81, 82]. Durvalumab, a humanised anti‐PD‐L1 monoclonal antibody, was evaluated in multiple clinical trials alone or in combination with tremelimumab and/or TKIs such as sorafenib in HCC patients. As a result, since durvalumab and tremelimumab combination therapy or durvalumab monotherapy showed significant survival improvement in HCC patients, in the first edition of the National Comprehensive Cancer Network guidelines for liver cancer in 2022, durvalumab was recommended as first‐line category 1 for treating advanced HCC [83, 84]. However, a major concern in HCC patients is the potential for viral outbreaks in hepatitis C virus (HCV) and hepatitis B virus (HBV)‐related cases. Nevertheless, in the CheckMate 040 clinical trial (Table 1), chronic HBV patients did not show any hepatitis flare [99, 100]. In fact, HBV regulates PD‐L1 expression in chronic hepatitis B via the phosphorylated signal transducer and activator of transcription 3 (pSTAT3): Sal‐like protein 4 (SALL4)‐microRNA‐200c (miR‐200c) axis in HBV‐associated HCC [101]. In contrast, Il27rα (also called WSX1) reduces T cell exhaustion by enhancing PD‐L1 degradation through the PI3Kδ/AKT/glycogen synthase kinase 3 beta (GSK3β)/PD‐L1 pathway [102]. Moreover, HCC cells may increase PD‐L1 expression via the B‐lymphocyte‐induced maturation protein‐1 (BLIMP1)‐ubiquitin‐specific protease 22 (USP22)–specificity protein 1 (SP1) axis, leading to exhaustion of CD8^+^ TILs. Therefore, HCC patients with high tumoral BLIMP1 expression exhibit remarkable responses to anti‐PD‐1 mAb therapy [103]. A novel hypothesis suggests that increasing PD‐L1 expression may enhance the therapeutic efficacy of anti–PD‐1 treatment. For instance, PD‐L1 induction via the IFNG/E1A binding protein p300 (p300)/myocyte enhancer factor 2D (MEF2D) mediated activation of the *CD274* gene (encodes PD‐L1) or targeting sirtuin 7 (SIRT7) that deactivates MEF2D improves sensitivity to PD‐1 blockade in HCC cells [104]. Moving forward, a better understanding of how the PD‐1/PDL‐1 axis derives T‐cell exhaustion will be essential for developing more effective immunotherapeutic modalities in this highly immunosuppressive cancer.

**TABLE 1:** Clinical trials on HCC patients targeting T cell exhaustion with different therapeutic approaches.

Medication (s)	Trial's phase	Clinical trials' ID	Target group	Key clinical outcomes	Mechanism of action/rationale	Toxicity/resistance notes	References
Nivolumab + ipilimumab	1/2	CheckMate040 (NCT0165887)	Advanced HCC patients previously treated with sorafenib	Objective response rate (ORR) was more than 30%, combination therapy led to high overall survival (OS) rates and had manageable safety profile	Nivolumab: anti‐programmed cell death protein 1 (anti‐PD‐1); ipilimumab: anti‐ cytotoxic T‐lymphocyte–associated protein 4 (anti‐CTLA‐4). Dual inhibition can synergize by expanding T cell activation and reducing regulatory T cell activity	Nivolumab and ipilimumab combination therapy showed manageable safety, promising, and durable responses	[85]
Atezolizumab + bevacizumab vs. sorafenib (1st. line)	3	IMbrave150 (NCT03434379)	Unresectable HCC patients with systemic treatment‐naïve	Median OS was 19.2 months for Atezolizumab plus Bevacizumab, and 13.4 months for sorafenib only group. The median progression‐free survival (PFS) was 6.9 and 4.3 months in the respectively, for the same treatment groups. The clinical outcomes showed efficacy for atezolizumab and bevacizumab combination therapy in patients with stage B disease based on Barcelona clinic liver cancer (BCLC)	Atezolizumab: anti‐ programmed death‐ligand 1 (anti‐PD‐L1), bevacizumab: anti‐ vascular endothelial growth factor (anti‐VEGF). Combination of these two factors aims to improve immune infiltration by normalising vasculature and reducing immunosuppressive VEGF signalling [86]	The most common treatment‐related adverse events with atezolizumab plus bevacizumab were proteinuria in 29%, hypertension in 28%, increased aspartate aminotransferase (AST) in 16%, and fatigue in 16% of the patients. In the sorafenib treated group, palmar‐plantar erythrodysesthesia syndrome in 48% and diarrhoea in 44% of the patients were the most common observed side‐effects	[87]
Pembrolizumab or placebo + Best Supportive Care (2nd line)	3	KEYNOTE‐240 (NCT0270240)	Previously treated advanced HCC, BCLC Stage C or B disease not amenable to or refractory to locoregional therapy	Median OS was 14.2 months in the pembrolizumab arm vs. 12.5 months in the placebo arm. The estimated percentage of alive participants at 12, 24, and 36 months was 56.0%, 31.2%, and 21.4%, respectively, in the pembrolizumab arm and 50.7%, 22.8%, and 9.8%, respectively, in the placebo arm	Pembrolizumab: anti‐PD‐1 reactivates exhausted T cells by blocking PD‐1–PD‐L1 interaction	Pembrolizumab had manageable adverse events profile	[88]
Durvalumab + tremelimumab (single tremelimumab regular interval durvalumab exposure, STRIDE) vs. durvalumab vs. Sorafenib (1st line)	3	HIMALAYA (NCT03298451)	Unresectable HCC patients who had not been previously treated with systemic therapy	The survival rates at 18, 24, 36, and 48 months were 47.4%, 39.6%, 24.7%, and 19.3%, respectively, in the durvalumab arm. ORR for STRIDE, Durvalumab, and Sorafenib were 51.5%, 53.3%, and 15.6%, respectively	Tremelimumab: anti‐CTLA‐4, durvalumab: anti‐PD‐L1 CTLA‐4 blockade primes immune activation; PD‐L1 blockade sustains effector function	Serious treatment‐related adverse effects, including death, occurred in 17.5%, 8.5%, and 9.6% of the patients, in STRIDE, Durvalumab, and Sorafenib arms, respectively. There were no new serious treatment‐related adverse events (AEs) in primary analysis of STRIDE	[89]
Relatlimab + nivolumab	2	NCT04567615	Advanced HCC patients who have never underwent immunotherapy therapy, after treatment with tyrosine kinase inhibitors	Early data showed promising synergy which previously hypothesized, however, no mature OS/PFS published yet. Based on preclinical data dual targeting of lymphocyte activation gene 3 (LAG‐3) by relatlimab, and PD‐1 by nivolumab may empower potential synergistic pathways to activate T‐cell and promote immune response	Relatlimab: anti‐LAG‐3; nivolumab: anti‐PD‐1 Dual blockade may relieve T cell suppression via two inhibitory axes	Not reported yet	[90]
Cobolimab + dostarlimab	2	NCT03680508	BCLC Stage B or C	Complete and partial response, stable disease, and disease progression have been seen in 1, 5 (ORR 46%), 3 (ORR23%), and 4 (ORR31%) patients, respectively. 57% of the patients with elevated alpha‐fetoprotein (AFP), demonstrated diminishing AFP level by greater than 50%. The interim results in advanced HCC patients showed promising clinical outcomes as first line treatment	Cobolimab: anti– T cell immunoglobulin and mucin domain–containing protein 3 (TIM‐3); Dostarlimab: anti–PD‐1 TIM‐3 is another exhaustion marker; blocking TIM‐3 in combination with PD‐1 may rescue more exhausted T cells	Early safety seems manageable; resistance could arise via compensatory inhibitory pathways (e.g., other checkpoint inhibitors) or immunosuppressive tumour microenvironment	[91]
Camrelizumab + rivoceranib vs. sorafenib (1st line)	3	CARES‐310 (NCT03764293)	Unresectable or metastatic HCC patients who had not received any systemic treatments previously	Median OS with camrelizumab‐rivoceranib in comparison with sorafenib was significantly extended, as follow: 22.1 months vs. 15.2 months, respectively. So, the combination therapy demonstrated that PFS and OS are statistically significant and clinically meaningful compared with sorafenib in unresectable HCC patients. Introducing a novel and effective first‐line treatment	Camrelizumab: anti–PD‐1; rivoceranib (Apatinib): vascular endothelial growth factor receptor (VEGFR2) tyrosine kinase inhibitor (TKI) Anti‐angiogenesis may improve immune access and patients' survival	Known immune checkpoint inhibitor (ICI) + TKI toxicity profile	[92]
Cabozantinib + atezolizumab vs. sorafenib	3	COSMIC‐312 (NCT0375579)	Previously untreated advanced HCC patients	Median OS was 16.5 months in the combination therapy group and 15.5 months (12.2–20.0) for the sorafenib group. Median progression‐free survival was 6.9 and 4.3, and 5.8 months for the combination therapy group, sorafenib, and single‐agent cabozantinib group, respectively. In advanced HCC patients, Cabozantinib, as a first‐line treatment in combination with atezolizumab could not improve OS in comparison with sorafenib, and PFS	Cabozantinib: TKI (c‐Met, VEGFR2, others); atezolizumab: anti–PD‐L1. TKI and ICI combination therapy can target tumour microenvironment (TME) and improve immune suppression status of the TME	No new signals; Possible escape pathways. Possible resistance mechanisms via alternative pathways, e.g., immune escape, TKI resistance can occur, so, safety consistent with known TKI + ICI toxicity profile	[93]
Lenvatinib + pembrolizumab vs. lenvatinib	3	LEAP‐002 (NCT03713593)	Patients aged 18 years or older with unresectable HCC, Child Pugh class A liver disease	Median OS was 21.2 and 21.7 months in Lenvatinib plus pembrolizumab versus lenvatinib plus placebo. Median progression‐free survival was 8.2 and 8 months in lenvatinib plus pembrolizumab versus lenvatinib plus placebo groups. So, the combination therapy did not demonstrate any improvement in OS and PFS in comparison with Lenvatinib and placebo (saline). The clinical trial's findings didn't provide any alterations in clinical practice	Pembrolizumab: anti–PD‐1; lenvatinib: multi‐kinase inhibitor (VEGFR1‐3, etc.) rationale: anti‐angiogenesis may improve immune infiltration	The most common treatment‐related grade 3–4 adverse events were as follow: Hypertension: 17% and 17% of patients in the Lenvatinib plus pembrolizumab group vs. Lenvatinib plus placebo group, respectively, increased AST 7% vs. 4%, and diarrhoea 6% vs. 4%, in the mentioned treatment groups. Treatment‐ related death: 1% in both treatment groups	[94]
Tremelimumab monotherapy	2	NCT01008358	HCC patients and chronic hepatitis C virus (HCV) infection	Partial response and disease control rate were 17.6% and 76.4%, respectively. Time to progression was 6.48 months. A significant drop in viral load was observed. Tremelimumab had good safety profile. Besides, the results showed its anti‐tumour and anti‐viral activity in HCV‐induced liver cirrhosis who developed advanced HCC. The latter effect was because of elevated specific ani‐HCV immune response	Anti–CTLA‐4 may enhance anti‐viral T cell responses, reduce viral load, and indirectly reduce HCC related complications	Immune‐related adverse events (IrAEs) were manageable; treatments' benefits may be limited, since compensatory immune suppression or T cell exhaustion via other pathways might happen	[95]
Nivolumab vs. *s*orafenib (1st line)	3	CheckMate459 (NCT02576509)	Histologically confirmed advanced HCC not eligible for surgery or locoregional treatment, or their disease had progressed after mentioned conventional therapies, with no previous systemic therapy for HCC, with class A Child‐Pugh	Median OS were 16.4 and 14.7 months with nivolumab and sorafenib treatment groups, respectively. OS did not alter significantly via first‐line nivolumab monotherapy comparing sorafenib, however, in HCC patients who TKIs are risky, nivolumab monotherapy can be considered as a therapeutic option	Nivolumab: anti‐PD‐1	Treatment‐related deaths as follow: four and one deaths in the nivolumab and sorafenib group, respectively. The most common grade 3 were palmar‐plantar erythrodysesthesia < 1%, patients in the nivolumab group vs. 14% of patients in the sorafenib group, aspartate aminotransferase increases 6% vs. 4%, and hypertension vs. 7%. Serious treatment‐related adverse events were reported in 12% patients receiving nivolumab and 11% patients receiving sorafenib	[82]
Pembrolizumab monotherapy (1st line, treatment‐naïve)	2	KEYNOTE‐224 (NCT02702414)	Advanced HCC patients without prior systemic therapy	Objective Response Rate (ORR): 16% [95% CI, 7–29]. Median duration of response (DOR): 16 months. Disease Control Rate (DCR): 57% Median OS was 17 months. The median PFS and median time to progression (TTP) were both 4 months	Pembrolizumab: anti‐PD‐1	Treatment‐related adverse events occurred in 16% of patients	[96]
Lenvatinib + KN046 (1st line)	2	NCT04542837	Advanced HCC: 92.7% of them were in BCLC stage C, and 96.4% had Child‐Pugh A liver function, and 50.9% participants had extrahepatic metastases	The median OS and 12‐month OS rate were 16.4 and 12 months, respectively. The ORR was 45.5%, and median PFS was 11.0 months	KN046: bispecific antibody, anti‐PD‐L1/CTLA‐4, lenvatinib: multi‐kinase inhibitor (VEGFR1‐3, etc.)	Serious adverse effects occurred in 30.9% of the participants, whom 14.5% of them considered treatment‐related adverse effects. Treatment‐related adverse effects happened in 100% of the patients, and in 47.3% of them these adverse effects were grade ≥ 3, 23.6% of the participants raised irAEs	[97]
Domvanalimab + zimberelimab	2	LIVERTI (NCT05724563)	Immunotherapy refractory advanced HCC patients receiving prior PD‐1/PD‐L1–based immunotherapy regimens, including combinations with VEGF, CTLA‐4, and LAG‐3 inhibitors	ORR 17.2% (95% CI 5.8%–35.8%); median PFS 4.4 months (95% CI 4.1–4.6 months)	Domvanalima: fragment crystallizable (Fc)‐silent anti‐TIGIT zimberelimab: anti–PD‐1	Treatment‐related adverse effects occurred in 55.2% of the patients, most of which were low‐grade and manageable. However, Serious or grade 3–4 treatment‐related adverse effects occurred in 10.3% of the patients, a rate considerably lower than the one reported with regorafenib	[98]

Abbreviations: AE, adverse event; AFP, alpha‐fetoprotein; AST, aspartate aminotransferase; BCLC, Barcelona Clinic Liver Cancer; CTLA‐4, cytotoxic T‐lymphocyte–associated protein 4; DCR, disease control rate; DOR, duration of response; Fc, fragment crystallizable; HCC, hepatocellular carcinoma; ICI, immune checkpoint inhibitor; irAE, immune‐related adverse event; KN046, bispecific antibody, anti‐PD‐L1/CTLA‐4; LAG‐3, lymphocyte activation gene 3; ORR, objective response rate; OS, overall survival; PD‐1, programmed cell death protein 1; PD‐L1, programmed death‐ligand 1; PFS, progression‐free survival; STRIDE, single tremelimumab regular interval durvalumab exposure; TIM‐3, T cell immunoglobulin and mucin domain–containing protein 3; TKI, tyrosine kinase inhibitor; TME, tumour microenvironment.TTP, time to progression; VEGF, vascular endothelial growth factor; VEGFR, vascular endothelial growth factor receptor.

### 4.2 CTLA‐4 Pathway

CTLA‐4 is another inhibitory receptor that plays a critical role in T cell exhaustion. Several inhibitory mechanisms related to CTLA‐4 have been proposed. CTLA‐4 inhibits the CD28 co‐stimulatory signal by competitively binding to its ligands CD80/86, thereby suppressing CD28/TCR signalling and causing a strong inhibitory signal for T cell activation [105]. CTLA‐4 signal transduction occurs via intracellular binding to phosphatases, such as protein phosphatase 2A (PP2A) and SHP‐2 [105, 106, 107]. It is also believed that CTLA‐4 interacts indirectly with tyrosine phosphatase SHP2 via PI3‐Kinase. Moreover, CTLA‐4 inhibits PKB activity, which is essential for T cells' proliferation and metabolic activation, likely through its association with PP2A [97, 108]. In addition, CTLA‐4 activates indoleamine 2,3‐dioxygenase (IDO), an enzyme that breaks down tryptophan. Tryptophan is an essential amino acid for T cell proliferation, and a decreased level of tryptophan leads to T‐cell exhaustion (Figure 2) [109, 110]. CTLA‐4 significantly blocks the activations of AP‐1 and NFAT, negative regulators of *CTLA‐4*, and has a lesser effect on NF‐κB [97]. CTLA‐4 is significantly overexpressed during T cell exhaustion, affects T_EFF_ cell cytotoxicity [97]. Tumour‐specific‐CD8^+^ T‐cells derived from HCC exhibit elevated expression of T cell exhaustion markers, including CTLA‐4, and lower levels of cellular proliferation and effector cytokine production (Figure 2) [111]. The first CTLA‐4‐blocking agent used in patients with HCC was tremelimumab, which demonstrated promising antitumor efficacy and an acceptable safety profile [95]. Morihara et al. reported that anti‐CTLA‐4 therapy enhances infiltration of IFN‐γ‐producing T‐helper 1 (Th1) cells, effectively attenuating HCC tumour growth, possibly through induction of cell cycle arrest and apoptosis [112]. Anti‐CTLA‐4 inhibits CD80/CD86 downregulation in dendritic cells (DCs) mediated by regulatory T cells, leading to increased cytokine secretion in DCs [113]. FDA approved the combination of immune checkpoint inhibitors, nivolumab (anti‐PD‐1) and ipilimumab (anti‐CTLA‐4) as a second‐line treatment for HCC based on the results of KEYNOTE‐224 and CheckMate 040 [85, 99, 114]. CTLA‐4 acts in concert with other inhibitory receptors [97]. Recently, the safety and efficacy of the bispecific anti‐PD‐L1/CTLA‐4 antibody KN046 in patients with advanced HCC were evaluated in a phase II trial (NCT04542837). Results showed an acceptable safety profile and promising efficacy in advanced metastatic HCC (Table 1) [115]. Taken together, additional studies are required to elucidate the mechanisms by which CTLA‐4 regulates immune responses, to clarify how its blockade exerts antitumor effects, and to develop more effective strategies for HCC treatment.

### 4.3 LAG‐3

LAG‐3, also known as CD223, is another inhibitory receptor expressed on the surface of T cells contributing to T cell exhaustion. Upon TCR or cytokine stimulation, IL‐3, IL‐7, IL‐12, and LAG‐3 expressions are upregulated and negatively regulate TCR signal transduction. At the immunological synapse between an APC and a T cell, LAG‐3 binds to major histocompatibility complex class II (MHCII), inhibiting TCR signalling during early activation. It prevents the activation of TFs such as NFAT and STAT‐5, leading to diminished cytokine production (e.g., IL‐2) and impaired proliferation [116, 117, 118]. LAG‐3 expression may be regulated by metalloproteases, a disintegrin and metalloproteinase domain‐containing protein (ADAM10/17), which cleaves LAG‐3 and converts it into a soluble form [116]. Elevated serum LAG‐3 levels are correlated with lower response rates after transarterial chemoembolization in patients with HCC [119]. Fibrinogen‐like protein 1 (FGL‐1) has been identified as another LAG‐3 ligand, and blockade of the FGL‐1‐LAG‐3 pathway may activate antitumor immunity and suppress tumour progression. Indeed, LAG‐3 expression is considered an unfavourable prognostic biomarker in HCC. A novel LAG‐3‐neutralising antibody, M234, inhibits both MHC‐II and FGL‐1 binding to LAG‐3. An in vitro study demonstrates that M234 significantly enhances IFN‐γ secretion in activated PBMCs. Consistently, in vivo experiments in a mouse model of hepatocellular carcinoma xenografts show that CAR‐T cells exhibit markedly improved therapeutic efficacy (Figure 2) [120]. Intriguingly, the transcriptional signature of exhausted CD8^+^ T cells correlates more strongly with CD8^+^LAG‐3^+^ cells than with CD8^+^PD‐1^+^ cells. A higher density of CD8^+^LAG3^+^ cells predicts better responses to anti‐PD‐1/PD‐L1 therapies, with more prolonged progression‐free and overall survival compared to CD8^+^PD‐1^+^ cells [121]. Indeed, PD‐1^+^ T cells are a heterogenous population containing both activated and exhausted T cells; however, PD‐1^+^/LAG‐3^+^ T cells are indicators of truly exhausted T cells [122]. Since LAG‐3 and PD‐1/PD‐L1 may act synergistically, combination therapy with anti‐LAG‐3 and anti‐PD‐L1 mAbs may represent a promising approach for HCC patients with high LAG‐3 and low PD‐L1 expression [123]. Nonetheless, clinical data show that tebotelimab, a PD‐1/LAG‐3 bispecific antibody, exhibits similar anti‐tumour activity in advanced HCC patients regardless of prior PD‐1/PD‐L1 therapy [124]. This may be partly due to the limited number of TILs in the TME. Treatment with a STING agonist in combination with a PD‐1/LAG‐3 antibody transitioned an immunosuppressive TME to an inflamed one, enhancing antitumor efficacy in a murine HCC model [125]. These findings highlight the need to study the therapeutic strategies that increase TILs in combination with immunotherapies to enhance ICI efficacy in HCC patients.

### 4.4 TIM‐3

TIM‐3 is extensively upregulated in TILs, resulting in T‐cell exhaustion during liver cancer progression [126]. TIM‐3, a type I transmembrane glycoprotein, is linked with suppression of anti‐tumour activity, poor prognosis, and tumour progression in HCC [127]. TIM‐3 has emerged as a potential prognostic biomarker in several cancers, including HCC, and may be a promising immunotherapy target [126, 128]. There are four known ligands for TIM‐3: galectin‐9 (Gal‐9), carcinoembryonic antigen‐related cell adhesion molecule 1 (CEACAM1), phosphatidylserine (PtdSer), and high‐mobility group box‐1 protein (HMGB1). In HCC, Kupffer cells express the highest level of Gal‐9 [129]. When TIM‐3 binds to Gal‐9, tyrosine residues in its cytoplasmic tail become phosphorylated, leading to the release of HLA‐B–associated transcript 3 (BAT3) and facilitating recruitment of the tyrosine kinase Fyn. Fyn then mediates the C‐terminal phosphorylation of LCK, thereby inhibiting its catalytic activity. As a result, T cell proliferation and cytokine production, such as IL‐2, tumour necrosis factor (TNFα), and INF‐γ, are suppressed [130, 131]. Inhibition of the TIM‐3/Gal‐9 signalling pathway restores T cell proliferation and effector function in HCC (Figure 2) [129]. In chronic hepatitis, TIM‐3 overexpression on the surface of CD4^+^ and CD8^+^ T cells decreases cytokine production; however, blocking TIM‐3 signalling significantly enhances the proliferation and cytokine secretion of CD8^+^ T cells in response to HBV antigen peptides. Additionally, TIM‐3 expression is enhanced with increasing severity of chronic hepatitis B [132]. Clinical data show that HCC patients with high CD8 levels and low TIM‐3 expression display better overall survival. Indeed, CD8^+^ T‐cell frequency and TIM‐3 expression are prognostic biomarkers for HCC [133]. Moreover, TIM‐3 is co‐upregulated with PD‐1 in CD4^+^ and CD8^+^ T cells from HCC samples. In preclinical HCC models, dual blockade of TIM‐3 and PD‐1 enhances antitumor immunity more effectively than single checkpoint blockade [134]. Therefore, restoring exhausted T cells by suppressing TIM‐3 alone or in combination with PD‐1 could be a promising therapeutic strategy and could be a breakthrough in liver cancer immunotherapy [133, 134].

### 4.5 ITIM Domain (TIGIT)

TIGIT is an inhibitory T cell receptor that limits T cell functions and adaptive immune responses during cancer immune evasion. Its ligands include CD155, CD112, CD113, and Nectin‐4, which are expressed on APCs and at higher levels on malignant cells, including HCC [98, 135, 136, 137]. Ligand‐receptor binding triggers phosphorylation of TIGIT's cytoplasmic tail leading to inhibition of downstream signalling pathways like PI3K‐Akt and MAPK, ultimately inhibiting T‐cell function (Figure 2) [138]. Additionally, TIGIT signalling directly downregulates CD3ε chain, TCR α‐chain, and PLCγ1 expression, thereby suppressing T_EFF_ cells' function. In summary, the TIGIT signalling pathway prevents both innate and adaptive anti‐tumour immune responses in the TME [139].

Transcriptomic analysis of clusters of CD8^+^ T‐cell subsets (naïve, functional effector, and exhausted; tumour‐specific CD8^+^ T cells) from the murine liver cancer model shows TIGIT upregulation during T‐cell exhaustion [140]. In another preclinical study, CD155 overexpression in HCC cell lines induces TIGIT up‐regulation in CD8^+^ T cells, decreasing secretion of INF‐γ, TNF‐α, IL‐17A, and increasing IL‐10. They also showed that TIGIT blockade or CD155 knockdown partially restores CD8^+^ T cell effector function [141]. In a recent study, clinical data from 140 HCC patients show that elevated TIGIT expression on T cells correlates with increased tumour volume, advanced stage, higher regulatory T cell proportion, elevated serum IL‐6 and IL‐10, and lower IFN‐γ concentrations (Figure 2) [142]. Consistently, Duan et al. reported that in patients with HCC, the poor tumour differentiation is positively correlated with higher TIGIT and CD155 expression [137]. Ge et al. demonstrated that ex vivo dual blockade of TIGIT and PD‐1 improved proliferation, cytokine production, and cytotoxicity of CD8^+^ T cells compared with single PD‐1 blockade in mononuclear leukocytes isolated from HCC patients [143]. Co‐downregulation of PD‐L1 and CD155 in Huh‐7 cells using downregulation of long non‐coding RNAs‐ colon cancer–associated transcript 1 (CCAT‐1), metastasis‐associated lung adenocarcinoma transcript 1 (MALAT‐1), or H19, or co‐downregulation of PD‐1 and TIGIT by overexpression of miR‐944‐5p and miR‐105‐5p in PBMCs enhances the cytotoxic potential of these cells [144]. Early clinical data also suggest potential for this approach. HCC patients who failed prior treatment with anti‐PD‐1/L1 show anti‐tumour effects; however, more data and stronger responses are needed [145].

### 4.6 Wnt/β‐Catenin Signalling Pathway

The immunosuppressive Wnt/β‐catenin signalling pathway shapes the immune landscape in HCC. PD‐L1 is stabilised during β‐catenin activation, while inhibition of GSK3β prevents β‐catenin ubiquitination and degradation, leading to PD‐1 up‐regulation in cancer cells and T cell exhaustion. Therefore, activators of GSK3β can be utilised to decrease T cell exhaustion and enhance immunotherapy efficacy [146, 147]. Moreover, β‐catenin mutant HCC cells impair dendritic cells‐mediated recruitment of T cells, thereby reducing T cell infiltration and activity. Chemokine (C‐C motif) ligand 5 (Ccl5) restores the efficacy of anti‐PD‐1 therapy in HCC [148]. Wnt pathway blockade using anti‐frizzled receptor genes (FZD) antibody Vantictumab (OMP‐a8R5), in combination with Paclitaxel, shows promising results in patients with HER‐2 negative breast cancer [149]. A phase Ib clinical study in advanced or metastatic HCC used OMP‐54F28 in combination with sorafenib, targeting the FZD8‐Fc decoy receptor [150]. Indeed, the Wnt/β‐catenin signalling pathway plays a critical role in establishing an immunosuppressive TME in HCC through multiple mechanisms, primarily by inducing PD‐L1 and impairing T‐cell recruitment and activation, thereby promoting immune evasion and resistance to immune checkpoint blockade therapies. However, emerging strategies targeting them show potential to reverse immune exclusion and enhance immunotherapy efficacy. Thus, application of Wnt pathway inhibitors as part of combination therapy regimens might overcome resistance and improve clinical outcomes in HCC patients.

### 4.7 TGFβ Signalling Pathway

TGFβ, a well‐known immunosuppressive cytokine, plays a crucial role in regulating cell growth, differentiation, angiogenesis, and tissue homeostasis. In addition to its canonical pathway, SMAD signalling, TGFβ also activates the MAP kinase signalling pathway, which triggers ERK1/2 or TGFβ‐activated kinase 1 (TAK1) [151]. In vitro results show that TGFβ impairs T cell proliferation and activation by reducing IL‐2 production and promotes T cell apoptosis [152]. In HCC samples, there is a robust link between the TGFβ signature and the exhausted immune signature, indicating its role in immune regulation in liver cancer. Clinical studies on other solid tumours have also shown elevated levels of T_EX_ cells and TGFβ in cervical and bladder cancers [153, 154]. In haematopoietic tumours, it also significantly suppresses T_EFF_ cells' cytotoxic function by downregulating key proteins, such as perforin, granzymes, and cytotoxins, at the transcriptional level [152]. Additionally, TGF‐β, in cooperation with ATF1, down‐regulates IFN‐γ expression, thereby diminishing the anti‐tumour function of cytotoxic CD8^+^ T cells [155]. In fact, T cell exhaustion is partly a response to a TME rich in TGFβ, secreted by cancer, stromal, and even immune cells themselves in liver cancer [155], and other solid cancers such as bladder and breast cancer [151, 154]. Furthermore, phosphorylated p38, a downstream factor of the TGFβ signalling pathway, contributes to increased PD‐1 and TIM‐3 expression in HCC‐infiltrating CD8^+^ T cells [156]. Evidence indicates that TGFβ1 decreases cytotoxic capacity and increases apoptosis of T cells in the HCC model. These effects appear to be driven, at least in part, by TGFβ1's ability to upregulate PD‐1 and CTLA‐4 on T lymphocytes, possibly through activation of the calcineurin‐NFATc1 signalling pathway. These findings support the rationale for immunotherapeutic strategies targeting CTLA‐4 and TGFβ1 in HCC (Figure 2) [157]. Preclinical studies on other solid cancers, such as pancreatic ductal adenocarcinoma (PDAC) and breast cancer, have shown that selective inhibition of TGFβ signalling in T_EFF_ cells leads to tumour regression [151, 158]. Galunisertib, a transforming growth factor beta receptor type (TGFβRI) kinase inhibitor, enhances the efficacy of sorafenib in inhibiting cell growth and apoptosis in preclinical HCC models [155]. In a clinical trial involving patients with advanced HCC, Galunisertib shows a favourable safety profile and is associated with lower circulating alpha‐fetoprotein and TGFβ levels. The latter effect is considered a potential survival indicator, although further studies are needed to validate these results [159, 160]. Nonetheless, TGFβ is reported to trigger a self‐rescue program via the p‐STAT1‐leukocyte‐associated immunoglobulin‐like receptor 2 (LAIR2) axis, which suppresses the inhibitory receptor LAIR1. This pathway is effective under low or transient TGFβ, but ineffective under high or long‐term TGFβ. Therefore, pharmacological activation of STAT1 by TAK‐981 improves the CD8^+^ T cell function in HCC [156]. However, systemic blockade of the TGFβ signalling pathway does not enhance cytotoxicity because PD‐L1 is upregulated [151, 158]. Thus, inhibiting both pathways has the potential to act synergistically in promoting an anti‐tumour immune response [158].

## 5 Metabolic Reprogramming has a Major Role in Driving T Cell Exhaustion

**FIGURE 3:**
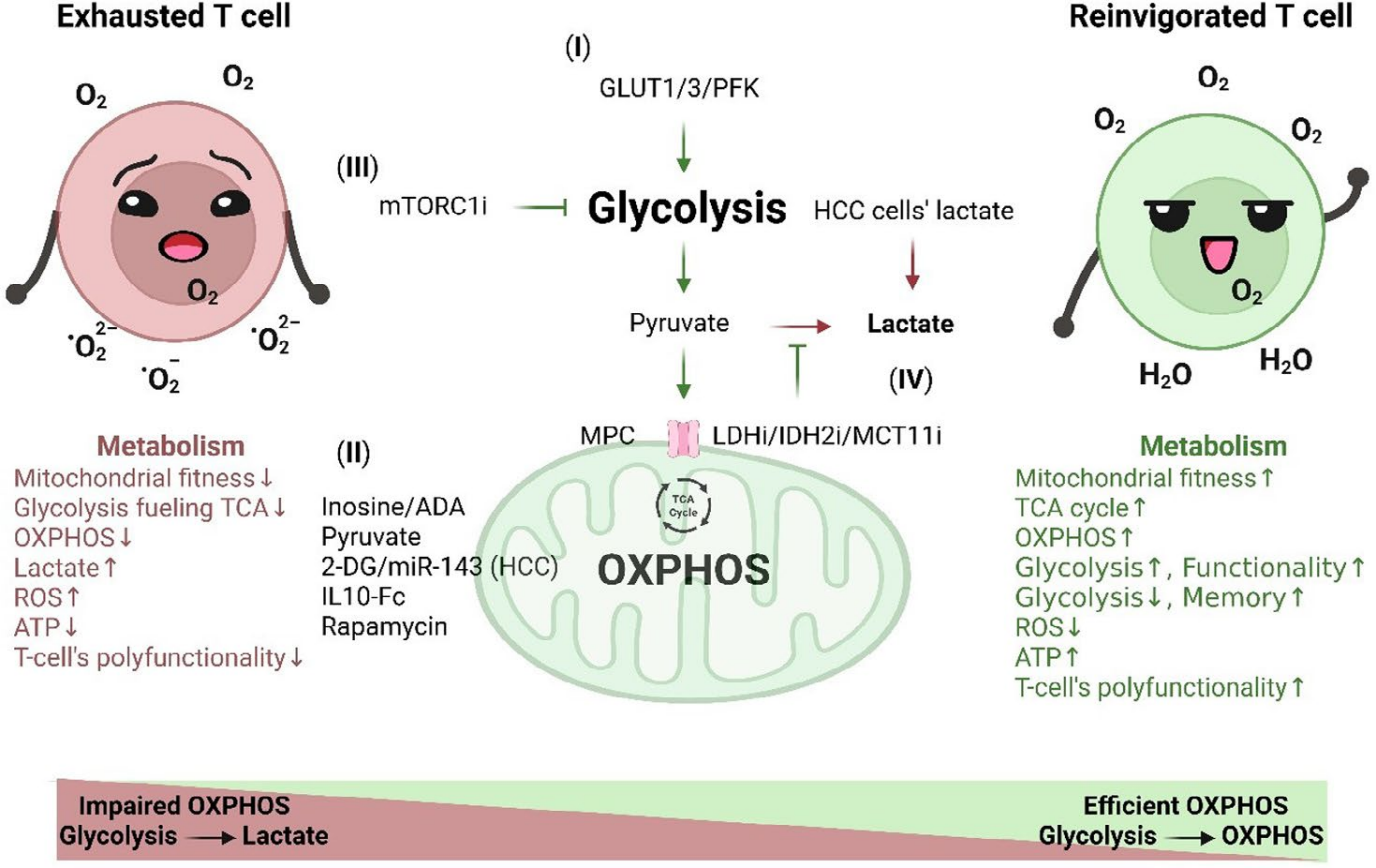
Targeting T‐cell metabolism could reinvigorate exhausted T cells. Metabolic reprogramming in exhausted T cells includes inefficient oxidative phosphorylation (OXPHOS) and glycolysis, which result in decreased energy production and increased reactive oxygen species (ROS). Therapeutic interventions to improve metabolism and mitigate exhaustion. (I) Glycolysis enhancement through different pathways reduced exhaustion and enhanced functionality of chimeric antigen receptor (CAR)/T cells, including overexpression of glucose transporter 1/3 (*GLUT1/3*), phosphofructokinase (*PFK*), and adenosine deaminase (ADA). (II) In contrast, glycolysis inhibition could augment chimeric antigen receptor (CAR)/T cell function by 2‐deoxy‐D‐glucose (2‐DG) or microRNA‐143 (miR‐143) in HCC. Supplementation of inosine/pyruvate/interleukin‐10 fused to fragment crystallizable (IL‐10/Fc) increases OXPHOS and decreases exhaustion. (III) Reducing glycolysis by mTORC1 inhibitor promotes memory phenotype at the expense of attenuating T cell polyfunctionality. Inhibiting anaerobic glycolysis could effectively ameliorate T cell exhaustion. (IV) Inhibiting lactate production by lactate dehydrogenase inhibitor (LDHi)/isocitrate dehydrogenase 2 inhibitor (IDH2) or lactate entrance by monocarboxylate transporter 11 blockade (MCT11) improves T‐cell exhaustion. Hepatic knock out of *Crif1* elevates glycolysis‐mediated lactate production in HCC cells, which promotes exhaustion markers expression and reduces polyfunctionality in T cells. Created in https://BioRender.com.

The differentiation and function of T cells are heavily dependent on metabolic pathways [161]. T_MEM_ cells use the fatty acid oxidation (FAO)‐derived TCA cycle and mitochondrial oxidative phosphorylation (OXPHOS) for long‐term survival. In contrast, T_EFF_ cells rely on aerobic glycolysis for rapid energy production [15, 162]. Within TME, nutrient deprivation and oxidative stress impair T cell metabolism. T_EFF_ cells are outcompeted for glucose and essential nutrients, leading to their exhaustion [36, 163]. During this transition, both OXPHOS and glycolysis become compromised [164]. Among the two exhausted subsets, T_PEX_ cells depend on OXPHOS for energy, whereas T_EX_ cells rely on inefficient OXPHOS and glycolysis to meet energy demands [15, 165]. Metabolic dysfunction reduces T cell polyfunctionality and proliferation. To address this, therapeutic strategies aimed at restoring metabolic fitness are under active investigation, with promising results in enhancing T cell responses [15, 164]. These approaches include glycolysis interventions, inhibition of anaerobic glycolysis, and enhancement of OXPHOS efficiency, as illustrated in Figure 3.

### 5.1 Glycolysis Interventions as an Approach to Reinvigorate T Cell Exhaustion

Some experiments have increased glycolytic capacity to restore normal glycolysis in T cells. Overexpression of glucose transporter 3 (*GLUT3*) or phosphofructokinase (*PFK*) increases adenosine triphosphate (ATP) production (by 56%), reduces exhaustion markers, and improves T cell functionality in melanoma models [166]. Similarly, glucose transporter 1 (*GLUT1*) overexpression decreases exhaustion markers (CD39, PD‐1) while boosting memory markers (CD62L, TCF1) in CAR‐T cells, leading to enhanced cytotoxicity and elevated IL‐2 and IFN‐γ production (Figure 3 I) [167]. Enhancement of glycolysis also inhibits GAPDH binding to the 3′‐UTR of IFN‐γ mRNA, preventing its translational downregulation [168]. Interestingly, Wang and his colleagues showed that under glucose‐deprived conditions, CD8^+^ CAR‐T cells use inosine instead of glucose without compromising cytotoxicity against tumour cells [163]. Overexpression of adenosine deaminase (ADA), an enzyme that converts adenosine to inosine, increases stem‐like memory populations while reducing T_EX_ cell subsets. Inosine supplementation further increases the effector function of CAR‐T cells [161]. Moreover, pyruvate supplementation overcomes glycolytic impairments and enhances CD8^+^ TIL activity [169]. Interleukin‐10 fused to fragment crystallizable (IL‐10/Fc) activates the TCA cycle and OXPHOS via the mitochondrial pyruvate carrier (MPC), thereby enhancing CAR‐T cell proliferation and tumour‐killing capacity in solid cancers [15, 18]. This was accompanied by promoting memory stem cell (T_SCM_) populations enriched in stemness and memory‐associated genes such as *Sell*, *Tcf7*, *Lef1*, *Il7r*, and *Ccr7* [18]. On the other hand, reducing glycolysis has also been shown to improve T cell exhaustion. Hypoxia‐inducible factor 1‐alpha (HIF‐1α) increases glycolysis but suppresses *Tcf7* and *Slamf6*, thereby driving T_PEX_ differentiation into T_EX_ cells during chronic infection. Inhibiting glycolysis with 2‐deoxy‐D‐glucose (2‐DG) increases OXPHOS, thereby enhancing CD19‐CAR‐T cell function in colorectal cancer [15]. Both 2‐DG and miR‐143 promote long‐lived memory phenotypes (CD44^+^, CD62L^+^), and upregulate memory‐associated genes (*Tcf7*, *Lef1*, and *Bcl6*), enhancing anti‐tumour responses in CD8^+^ T cells derived from healthy individuals [23, 170] and patients with breast and liver tumours (Figure 3 II) [171].

TCR/CD28 activation triggers PI3K/AKT/mTORC1/MYC and HIF1 pathways, promoting glycolysis, expansion, and cytokine production in T cells [164, 172]. Rapamycin‐mediated mTORC1 inhibition reduces glycolysis while enhancing adenosine monophosphate‐activated protein kinase (AMPK)/FAO. This intervention enhances energy production and expands memory CD8^+^ T cell populations, although recall responses are diminished [173]. Inhibiting mTOR also improves mitochondrial fitness by reducing mitochondrial depolarization and lowering glycolysis rate, promoting a memory phenotype at the expense of attenuating T cell polyfunctionality through reduced expression of PR domain zinc finger protein 1 (*Prdm1*), perforin (*Prf1*), and granzyme B (*Gzmb*) (Figure 3 III) [170, 174, 175]. Targeting mTORC1 with Vistusertib synergizes with ICIs and, in addition to augmenting memory T cell populations, enhances effector function and reduces exhaustion in colon cancer models [176]. The timing of mTOR inhibition is critical. Early treatment promotes stem‐like T cell proliferation, whereas late inhibition after exhaustion is established inhibits their differentiation into CX3CR1^+^TIM3^+^ transitory T_EFF_ cells. mTOR signalling is required for the differentiation of stem‐like T cells into these transitory T_EFF_ cells. The differentiation process is promoted by PD‐1 blockade; therefore, mTOR inhibition is essential for its effectiveness. These findings suggest two different strategies. In the first one, mTOR inhibition is administered before PD‐1 blockade to enhance its effect, as it increases the stem‐like repertoire without impairing differentiation into transitory T_EFF_ cells. In the second approach, mTOR activation is explicitly targeted to antigen‐specific CD8^+^ T cells simultaneously with PD‐1 blockade, thereby promoting their differentiation into transient T_EFF_ cells without compromising stem‐like T cell proliferation [177].

### 5.2 Anaerobic Glycolysis Inhibition Reverses T Cell Exhaustion

Inhibiting anaerobic glycolysis may effectively ameliorate T cell exhaustion. Lactate dehydrogenase inhibitors (LDHi) synergize with IL‐2 by fueling the TCA and OXPHOS pathways, thereby improving antitumor responses in melanoma models [178]. Inhibition of isocitrate dehydrogenase 2 (IDH2), a TCA cycle enzyme, by Enasidenib may also decrease LDH activity, at least in part via NCOR1‐mediated suppression of glycolytic gene expression [36]. Furthermore, combining LDHi with IL‐21 yields improved therapeutic outcomes. This combination not only reduces IL‐21‐induced exhaustion markers but also promotes a memory T cell phenotype, thereby enhancing the durability of anti‐tumour immunity [178]. Metabolites in the TME can also influence TIL exhaustion. CR6‐interacting factor 1 (CRIF1), a mitochondrial ribosome protein, contributes to the synthesis of OXPHOS polypeptides. Hepatic knock‐out of *Crif1* elevates glycolysis‐mediated lactate production in HCC cells, promoting exhaustion markers expression and reducing T‐cell polyfunctionality [179]. MCT11, a monocarboxylate transporter 11 (MCT11), enhances lactic acid uptake under chronic stimulation or hypoxia *in a* HIF‐1α‐dependent manner, thereby restricting T cell function. Blockade of MCT11 improves T_EX_ cells' polyfunctionality and enhances the efficacy of anti‐PD‐1 therapy in colorectal cancer models (Figure 3 IV). This intervention increases the response rate from 42% to 79%, correlating with the expansion of a T_PEX_ population marked by elevated *Tcf7* and MYB proto‐oncogene (*Myb*) expression [180].

## 6 The Role of Cellular Components Within the TME in Driving T Cell Exhaustion

The TME comprises diverse cell types, including tumour‐associated macrophage (TAM), myeloid‐derived suppressor cells (MDSCs), regulatory T cells (Tregs), and tumour endothelial cells (TECs), that can contribute to T cell exhaustion [181, 182, 183].

### 6.1 TAMs and MDSCs

TAMs play a crucial role in T cell dysfunction and exhaustion, as indicated by the correlation between TIM3^+^PD1^hi^ TILs and TAM presence [21], and by the spatial divergence between CD8^+^ T cells and TAMs in responsive HCC tumours [184]. TAMs facilitate CD8^+^ T cell recruitment and cause chronic activation‐mediated exhaustion. In vivo studies in melanoma and breast cancer models show that acute TAM depletion reduces exhaustion markers like PD‐1 and CD38 on TILs, reinforcing their contribution to T cell exhaustion [185]. Additionally, M2 macrophage markers, including transmembrane domain‐containing 7 (CMTM7), exhibit a positive correlation with various exhaustion markers in HCC samples [186]. Beyond cellular interactions, TAM‐derived secretome also contributes to CD8^+^ T cell exhaustion. M2 macrophage‐derived extracellular vesicles (EVs), enriched in miR‐21‐5p through Ubiquitin thioesterase ovarian tumour 1 (YOD1)/Yes‐associated protein 1 (YAP)/β‐catenin axis, induce exhaustion in CD8^+^ cells in HCC model [187]. Similarly, Hepa1‐6 cells‐derived EVs, enriched with miR‐146a‐5p, and regulated by SALL4, promote M2 polarisation. These educated macrophages further drive T cells exhaustion in HCC model [188]. Loss of *Xdh* gene promotes M2 polarisation in macrophages, which, through IDH3α/adenosine and kynurenic acid promotes CD8^+^ T cell exhaustion in the HCC TME [189]. Additionally, cyclooxygenase‐2 (COX‐2) upregulation in tumour cells enhances M2 polarisation and activates the TGF‐β pathway, leading to exhaustion of CD8^+^ T cells in preclinical HCC models [190].

Similarly, MDSCs impair anti‐tumour immunity by inhibiting T and natural killer cells while promoting Tregs within the HCC TME [191, 192, 193]. These immature myeloid cells support tumour progression through various mechanisms. S100A8/A9, a hallmark of MDSCs, upregulates C‐X‐C motif chemokine ligand (CXCL), contributing to CD8^+^ T cell exhaustion in gastric cancer [194]. Furthermore, MDSCs promote exhaustion markers' expression in CD8^+^ T cells through the CD84/Akt/Stat3/PD‐L1 axis [195].

### 6.2 Tregs

Tregs further contribute to T cell exhaustion through the secretion of IL‐10 and IL‐35. IL‐10 stimulates mature protein‐1 in B lymphocytes that inhibits CD28 tyrosine phosphorylation, thereby promoting CD8^+^ T cell exhaustion. IL‐35 increases the expression of PD‐1, TIM3, and LAG3 on T cells, facilitating immune evasion [196]. Kalathil et al. highlight the immunosuppressive roles of Tregs and MDSCs in HCC. Tivozanib, a tyrosine kinase inhibitor, reduces Tregs and MDSCs accumulation by blocking 9a receptor tyrosine kinase (RTK) (c‐Kit)/stem cell factor (SCF) signalling, thereby reversing tumour‐induced immune suppression via an inhibition of ERK2 phosphorylation [197]. Additionally, in vitro studies show that depletion of MDSCs, Tregs, and PD‐1^+^ exhausted T cells restore CD8^+^ T cell function and Gzmb production in HCC patient PBMCs [198]. Studies on hepatocellular carcinoma tissues have shown that CCR8^+^ Tregs are increased within the tumour microenvironment, while CD8^+^ T‐cell activity is reduced, leading to immune modulation and enhanced tumour growth and progression. In this study, targeting this Treg subset with an anti‐CCR8 antibody diminished their suppressive function and consequently restored and increased cytotoxic T‐cell activity [199].

### 6.3 TECs

A strong correlation is observed between TECs and exhausted CD8^+^ T cells within the TME. In murine HCC models, TEC injection leads to a reduction in CD8^+^ T cells and accelerated tumour progression. Next‐generation sequencing identified glycoprotein nonmetastatic melanoma protein B (GPNMB) as a key regulator of TEC‐mediated T cell exhaustion in subcutaneous HCC tumours [200]. In fibrosis models, liver sinusoidal endothelial cells (LSECs) exhibit high levels of immunosuppressive molecules such as PD‐L1, ICAM‐1, and H2‐Kb (MHCI). Under chronic liver injury, LSECs induce exhaustion markers, particularly TIGIT, while reducing cytokine production, surpassing dendritic cells in immunosuppressive effects [201]. Notably, TIGIT has emerged as a more reliable marker of T cell exhaustion in liver cancer than PD‐1, and combination therapy targeting both pathways demonstrates superior tumour control compared to PD‐1 blockade alone [140].

## 7 Concluding Remarks and Future Perspectives

The limited success of immunotherapy in solid tumours including HCC is primarily attributed to T cell exhaustion, a dysfunctional state that impairs antitumor immunity [202]. A deeper understanding of the mechanisms driving this state is essential not only to enhance the antitumor activity of endogenous T cells but also to improve the efficacy of CAR‐T cells. Preventing stable epigenetic changes in progenitor exhausted T cells is crucial to halting their progression toward terminal exhaustion. Moreover, modulating rather than completely suppressing the expression or function of key exhaustion regulators, such as TOX, and key signalling pathways, like inhibitory receptors, and restoring metabolic fitness may effectively limit T cell exhaustion in HCC. Furthermore, inhibiting the TME factors that drive exhaustion could similarly reinvigorate T cells.

Identifying reliable biomarkers predictive of immunotherapy response is pivotal for developing effective, personalised cancer treatments. Single‐cell RNA sequencing (scRNA‐seq) and computational models have enabled the identification of T cell exhaustion genetic signatures in both exhausted T cells and non‐immune cells within the pro‐exhaustion microenvironment [12, 203]. Additionally, spatial transcriptomic sequencing (ST‐seq) provided abundant information on the association between non‐immune cells and CD8^+^ T cells [184]. These data are used to predict responses to immunotherapies [184, 204]. Combining scRNA‐seq and ST‐seq should provide information about the genes in which cells contribute to T cell exhaustion or even to therapeutic response. Ultimately, identifying gene or protein signatures predictive of immunotherapy efficacy should guide the selection of HCC patients most likely to benefit from specific treatments, as demonstrated in a recent study by Ding et al. [205] on liver cancer. Moving forward, a deeper understanding of resistance and response mechanisms is essential for developing novel, more effective therapeutic strategies. Continued investigations on HCC samples to uncover valuable predictive biomarkers in the context of immunotherapy will pave the way in the fight against liver cancer.

## Nomenclature

**Genes**
*BACH2*
: BTB domain and CNC homology 2
*BATF*
: Basic leucine zipper ATF‐like transcription factor
*BCL6*
: B‐cell lymphoma 6 protein
*CCL5*
: Chemokine (C‐C motif) ligand 5
*CCR7*
: C‐C chemokine receptor 7
*CD27*
: Cluster of Differentiation 27 (also known as TNFRSF7, Tumour Necrosis Factor Receptor Superfamily Member 7)
*CD274*
: Cluster of differentiation 274. Gene encoding PD‐L1
*CRIF1*
: CR6‐interacting factor 1
*CTLA4*
: Cytotoxic T‐lymphocyte‐associated protein 4
*CXCL13*
: C‐X‐C motif chemokine ligand 13
*CXCR3*
: C‐X‐C motif chemokine receptor 3
*CXCR6*
: C‐X‐C motif chemokine receptor 6
*ENTPD1*
: Ectonucleoside triphosphate diphosphohydrolase 1
*EOMES*
: Eomesodermin
*ETS*
: Erythroblast transformation‐specific
*FOXO1*
: Forkhead box O 1
*FYN*
: FYN Proto‐Oncogene, SRC Family Tyrosine Kinase
*FZD*
: Frizzled receptor genes (e.g., FZD8)
*GLUT1/3*
: Glucose transporter 1/3
*GSK3β*
: Glycogen synthase kinase 3 beta
*GZMB*
: Granzyme B
*HAVCR2*
: Hepatitis A virus cellular receptor 2
*IFNG/IFN‐α*
: Interferon gamma/alpha
*IKZF2*
: IKAROS family zinc finger 2
IL‐10/Fc: Interleukin‐10 fused to fragment crystallizable
*IL12*
: Interleukin‐12
*IL‐2*
: Interleukin‐2
*IL2Rα*
: Interleukin 2 receptor alpha chain
*IL3*
: Interleukin‐3
*IL7*
: Interleukin‐7
*IL7R*
: Interleukin 7 receptor
*IRF4*
: Interferon regulatory factor 4
*KDM*
: Lysine demethylase
*KLRG1*
: Killer cell lectin‐like receptor G1
*Layn*
: Layilin
*LEF1*
: Lymphoid enhancer‐binding factor 1
*MEF2D*
: Myocyte enhancer factor 2D
*miR‐200c*
: MicroRNA‐200c
*MYB*
: MYB proto‐oncogene, transcription factor
*MYC*
: MYC proto‐oncogene, bHLH transcription factor
*MYO7A*
: Myosin VIIA
*NFAT*
: Nuclear factor of activated T‐cells
*NFATC1*
: Nuclear factor of activated T‐cells, cytoplasmic 1
*NR4A1*
: Nuclear receptor subfamily 4 group A member 1
*PDCD1*
: Programmed Cell Death 1 (encodes PD‐1)
*PFK*
: Phosphofructokinase
*PRDM1*
: PR domain zinc finger protein 1
*PRF1*
: Perforin 1
*SELL*
: L‐selectin (CD62L)
*SNAP47*
: Synaptosomal‐associated protein 47 kDa
*SPI1*
: Specificity protein 1
*TAK1*
: TGF‐beta Activated Kinase 1
*TBX21*
: T‐box transcription factor 21
*TCF7*
: T cell factor 7
*TIGIT*
: T cell immunoreceptor with Ig and ITIM domains
*TNFRSF9*
: Tumour necrosis factor receptor superfamily member 9
*TOX*
: Thymocyte selection‐associated high mobility group box
*VHL*
: Von hippel–lindau tumour suppressor
*WARS*
: Tryptophanyl‐tRNA synthetase

**Proteins**
ADA: Adenosine deaminase
AFP: Alpha‐fetoprotein
AKT/PKB: Protein kinase B
AMPK: Adenosine monophosphate‐activated protein kinase
AP‐1: Activator protein 1
ATF: Activating transcription factor
2B4: Natural killer cell receptor 2B4
BAT3: HLA‐B–associated transcript 3
BCL‐xL: B‐cell lymphoma‐extra‐large
BLIMP1: B‐lymphocyte‐induced maturation protein‐1
β‐catenin: Key effector in Wnt signalling
BTLA: B and T lymphocyte attenuator
CEACAM1: Carcinoembryonic antigen‐related cell adhesion molecule 1
CD3ε: CD3 epsilon chain
CD39: Ectonucleoside triphosphate diphosphohydrolase‐1
CD62L: L‐selectin
CMTM7: Transmembrane domain‐containing 7
CXCL: C‐X‐C motif chemokine ligand
CRIF1: CR6‐interacting factor 1
COX‐2: Cyclooxygenase‐2
c‐Kit: 9a receptor tyrosine kinase (RTK)
c‐FOS: FBJ murine osteosarcoma viral oncogene homologue
CSK: C‐terminal Src kinase
CTLA‐4: Cytotoxic T‐lymphocyte‐associated protein 4
DNMTs: DNA methyltransferases
DNMT1: DNA methyltransferase 1
DNMT3A: DNA methyltransferase 3A
EZH2: Enhancer of Zeste Homologue 2
EGFR: Epidermal growth factor receptor
FGL1: Fibrinogen‐like protein 1
FOXO1: Forkhead Box O1
FYN: Proto‐oncogene tyrosine‐protein kinase Fyn
Gal‐9: Galectin‐9
GPNMB: Melanoma protein B
GSK3β: Glycogen synthase kinase 3 beta
HDAC8: Histone deacetylase 8
HBO1: Histone acetyltransferase binding to origin recognition complex subunit 1 (ORC1)
H3K27ac: Histone H3 acetylated at lysine 27
H3K4me3: Histone H3 trimethylated at lysine 4
HIF1α: Hypoxia‐inducible factor 1‐alpha
HMGB1: High‐mobility group box 1
IDH2: Isocitrate dehydrogenase 2
IFN‐γ/α: Interferon gamma/alpha
IRF9: Interferon regulatory factor 9
ITAM: Immunoreceptor tyrosine‐based activation motif
ITIM/ITSM: Immunoreceptor tyrosine‐based inhibitory/switch motif
KDM6B: Histone H3 lysine 27 (H3K27) demethylase
LAIR1: Leukocyte‐associated immunoglobulin‐like receptor 1
LAT: Linker for activation of T cells
LAG‐3: Lymphocyte activation gene 3
LCK: Lymphocyte‐specific protein tyrosine kinase
LDHi: Lactate dehydrogenase inhibitors
LEO1: A subcomplex of the RNA polymerase II‐associated factor 1 complex (Paf1C)
LAIR: Leukocyte‐associated immunoglobulin‐like receptor
MEK: Mitogen‐activated protein kinase kinase
MHCI/II: major histocompatibility complex class I/II
MCT11: Monocarboxylate transporter 11
MPC: Mitochondrial pyruvate carrier
mTOR: Mammalian target of rapamycin
MYC: Myc‐proto‐oncogene family
NFAT/NFATc1: Nuclear factor of activated T cells/Nuclear factor of activated T cells, cytoplasmic, calcineurin dependent 1 also known as NFAT2
NF‐κB: Nuclear factor kappa‐light‐chain‐enhancer of activated B cells
Paf1: Polymerase‐associated factor 1
P300: E1A binding protein p300
PD‐1: Programmed cell death protein 1
PD‐L1: Programmed death‐ligand 1
PI3K: Phosphoinositide 3‐kinase
PKC: Protein kinase C
PLC‐γ1: Phospholipase C gamma 1
pMHC: peptide‐major histocompatibility complex
PP2A: Protein phosphatase 2A
PtdSer: Phosphatidylserine
SALL4: Sal‐like protein 4
Sap130/Sin3A: Sin3A associated protein 130/Switch‐independent 3A transcription regulator
SCF: Stem cell factor
SDH: Succinate dehydrogenase
SIRT7: Sirtuin 7
SLP‐76: SH2 domain‐containing leukocyte protein of 76 kDa
STAT: Signal transducer and activator of transcription
TGFβ: Transforming growth factor
TGFβRI: Transforming growth factor beta receptor type I
TIM‐3: T cell immunoglobulin and mucin‐domain containing‐3
TIGIT: T cell immunoreceptor with Ig and ITIM domains
TNFα: Tumour necrosis factor
USP22: Ubiquitin‐specific peptidase 22
ZAP‐70: Zeta‐chain‐associated protein kinase 70
YAP: Yes‐associated protein 1
YOD1: Ubiquitin thioesterase ovarian tumour 1 (OTU1)
Wnt: Wingless/integrated signalling pathway
WSX1: Interleukin‐27 receptor subunit alpha (Il27rα)
ERK/ERK1/2: Extracellular signal‐regulated kinases/1/2
SHP‐1/2: Src homology 2 domain‐containing phosphatase 1/2
CD80/CD86: B7‐1/B7‐2 co‐stimulatory molecules
ADAM10/17: A disintegrin and metalloproteinase domain‐containing proteins

**Others**
ATAC‐seq: Transposase‐accessible chromatin with high‐throughput sequencing
CAR: Chimeric antigen receptor
FAO: Fatty acid oxidation
FDA: The United States food and drug administration
GPC3: Glypican‐3
HBV: Hepatitis B virus
HCC: Hepatocellular carcinoma
HCV: Hepatitis C virus
ICI: Immune checkpoint inhibitor
mAbs: Monoclonal antibodies
MDSCs: Myeloid‐derived suppressor cells
MTA: 5‐methylthioadenosine
ORCs: Open chromatin regions
OXPHOS: Oxidative phosphorylation
PBMCs: Peripheral blood mononuclear cells
PDAC: Pancreatic ductal adenocarcinoma
SAM: S‐adenosylmethionine
scRNA‐seq: Single‐cell RNA sequencing
TAM: Tumour associated macrophage
Taz: Tazemetostat
TCA: Tricarboxylic acid
TCR: T cell receptor
TEC: Tumour endothelial cell
T_EFF_
: Effector T cells
T_EX_
: Exhausted T cells
TILs: Tumour‐infiltrating lymphocytes
TME: Tumour microenvironment
T_MEM_
: Memory T cells
T_PEX_
: Progenitor exhausted T cells
Tregs: Regulatory T cells

## Author Contributions

Kosar Nouri and Massoud Vosough contributed to the conception, design, and drafting of the manuscript. Kosar Nouri, Homeyra Seydi, Negar Asadollahei, and Yasamin Sharif were involved in data collection and manuscript writing. Negar Asadollahei and Kosar Nouri prepared the figures. Mahsa Salehi edited the full manuscript. Mustapha Najimi, Mehrnaz Mesdaghi, and Massoud Vosough provided scientific editing. All authors reviewed and approved the final version of the manuscript for submission.

## Funding

The authors have nothing to report.

## Ethics Statement

The authors have nothing to report.

## Conflicts of Interest

The authors declare no conflicts of interest.

## Data Availability

Data sharing not applicable to this article as no datasets were generated or analysed during the current study.
